# Poultices as biofilms of titanium dioxide nanoparticles/carboxymethyl cellulose/Phytagel for cleaning of infected cotton paper by *Aspergillus sydowii* and *Nevskia terrae*

**DOI:** 10.1007/s11356-023-30353-7

**Published:** 2023-10-21

**Authors:** Maisa M. A. Mansour, Mohamed Z. M. Salem

**Affiliations:** 1 Conservation Department, Faculty of Archaeology, Cairo University, Giza, 12613 Egypt; 2 Forestry and Wood Technology Department, Faculty of Agriculture (El-Shatby), Alexandria University, Alexandria, 21545 Egypt

**Keywords:** Poultices, Antimicrobial activities, Molecular identification, Mechanical properties, SEM–EDX, TEM, Manuscript preservation

## Abstract

In this study, TiO_2_ nanoparticle (TiO_2_NP)-coated film was produced to protect manuscripts against microorganisms using ecofriendly benign materials. As a result, a simple method was created that uses poultice biofilm made of carboxymethyl cellulose (CMC) and Phytagel plant cell (PGP) loaded with TiO_2_NPs to preserve manuscripts against microbes in an environmentally responsible way. Three volumes (1, 2, 4 mL) of TiO_2_NPs were put into a biofilm combination to produce the poultices known as CMC/PGP/TiO_2_-1, CMC/PGP/TiO_2_-2, and CMC/PGP/TiO_2_-3. The synthesized TiO_2_NPs were nearly spherical in shape, small in size (98 nm), and stable (zeta potential value − 33 mV). The results showed that the unique deposition of TiO_2_NPs on the biofilm surface gave the produced films loaded with TiO_2_NPs a rough structure. The highest values of mechanical characteristics were determined to be in CMC/PGP/TiO_2_-1 with values of 25.4 g, 6.6 MPa, and 11.4%, for tensile strength, elongation at break, and tear strength, respectively. Based on molecular identification, the fungus *Aspergillus sydowii* and the bacterium *Nevskia terrae*, with accession numbers MG991624 and AB806800, respectively, were isolated and identified from an antiquated manuscript formed from cellulosic fibers. Before the experiments, the produced cotton paper samples were aged, and then, one group was infected for 6 months by *A. sydowii* and the second group with *N. terrae*. Following the preparation of a CMC/PGP biofilm loaded with various volumes of TiO_2_NPs, poultices were applied to infected cotton paper in order to clean it. The infected cotton paper was placed inside the sandwich-like poultices that were created. The poultice CMC/PGP/TiO_2_-2 demonstrated potential for preventing the growth of *A. sydowii* and *N. terrae*-infected cotton paper, when the fibers were saved, cleaned, and coated with CMC/PGP/TiO_2_-2 after absorbing the fungus and the bacterium and exhibiting exceptional antimicrobial activities. Finally, the novel biofilms have demonstrated their capacity to lessen microbial contamination of cotton paper. In order to generalize the usage of these poultices, it is also advised that they be produced on a large scale and tested on a variety of organic materials in the future.

**Graphical Abstract:**

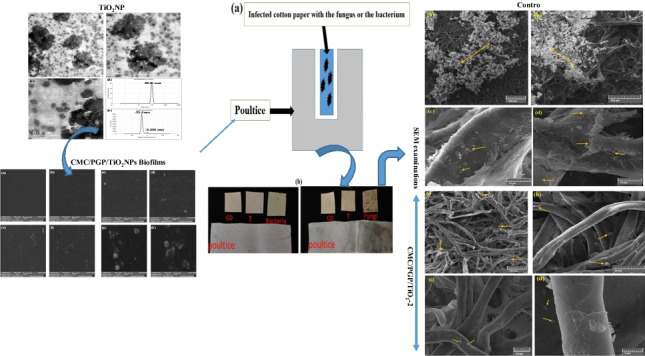

**Supplementary Information:**

The online version contains supplementary material available at 10.1007/s11356-023-30353-7.

## Introduction

Biodeterioration of historical manuscripts such as paper, photographic parchment, albumin prints, or papyrus or manufactured from organic materials becomes a significant societal and economic issue, once the spores and/or vegetative cells of microorganisms are found on the surface (Sterflinger and Piñar 2013; Ts et al. 2015; Borrego et al. 2018; Kraková et al. 2018; Eldeeb et al. 2022; Afifi et al. 2023; Mansour et al. 2023). Cultural asset protection is severely compromised by the potential for significant biodeterioration caused by some microbial metabolic activities on organic materials, which results in constant microbial deterioration and distortion (Caneva et al. 2008; Kwaśna et al. 2020; Branysova et al. 2022).

The microorganisms that degrade cellulose, create colors, and produce acids also contribute to biofouling, which itself is made up of numerous components including insect feces, in addition to harming aesthetics (Borrego et al. 2008; Guiamet et al. 2011; Di Carlo et al. 2022). Fungi were shown to be involved in the biodeterioration of antique etchings on different paper materials. This was corroborated by the finding that some fluorescence, which is activated by localized moisture accumulation, may originate from sulfate-containing fungus colonies (Zotti et al. 2008). *Aspergillus*, *Cladosporium*, and *Penicillium*-related fungi and proteolytic bacteria have been identified in the materials stored at the Historical Archive of the Museum of La Plata, Argentine, and at the National Archive of the Republic of Cuba (Guiamet et al. 2011).

Preventing biodeterioration and, as a result, protecting cultural heritage objects require accurate identification of the bacteria and fungi that are present. Because they create color and pigment, bacteria and fungi are a very diverse group of microbes, and as a result, their metabolic activities harm cultural artifacts, where fungi are more hardy than bacteria (Branysova et al. 2022). According to a decayed archaeological manuscript from the seventeenth century (1677 A.D.), *Bacillus subtilis* strain (B3) and *Penicillium chrysogenum* strain (F9) were found to have the highest levels of cellulolytic activity among the identified bacterial and fungal strains (Fouda et al. 2019). By causing oxidation and hydrolysis, *A. sydowii*, *A. flavus*, and *P. chrysogenum* destroyed albumen prints. These microorganisms can grow on model albumen silver prints, affecting the binder and potentially transferring to the paper fibers (Eldeeb et al. 2022).

Maintaining and preserving antiquities is becoming more challenging due to the increase in the handling and use of various artifacts in unfavorable environmental conditions (Okpalanozie et al. 2018). It is essential to clean and secure the papers before putting them inside the cases to reduce the possibility of microbial growth in the future under adverse environmental conditions or in the event of water hazards. Therefore, a number of conservation techniques were used to save the cultural heritage including certain traditional techniques (Tyagi 2023).

In some libraries, the wooden planks used to support the bundle of manuscripts are constructed of neem wood, which can fend off termites because they are vulnerable to insect assault. A dried leaf from a neem tree was inserted between papers to get rid of the booklice (Patidar and Soni 2016). Additionally, it has been observed that many libraries are implementing modern preservation strategies, such as microfilms, microfiches, and digitization (Idoko and Onwudinjo 2021; Mandal et al. 2023). Furthermore, 2-mM silver nanoparticle (AgNP) was a successful green conservation method for materials containing cellulose to inhibit entire microbial growth on paper models (Fouda et al. 2019).

Biofilm-based natural polymers are eco-friendly materials that can protect manuscripts against the development of microbes. Polymers like polyvinyl alcohol, poly(lactic acid), polypropylene, and poly(glycolic acid) have been used in the production of polymeric membranes, because of their biocompatibility, recyclability, and high mechanical strength (Santacruz et al. 2015). Edible biopolymer films can be produced by combining proteins (such as gelatin, casein, wheat gluten, and zein) and polysaccharides (such as alginate, starch, and chitosan) (Morales-Jiménez et al. 2020; Santacruz et al. 2015).

In the past, cellulosic or clay-based wet poultices were used to remove soluble salts (Ottosen and Christensen 2012; Feijoo et al. 2015). The mechanical properties of paper fibers can be improved by tightening the connections between the cellulose fibers (Graupner et al. 2009; Lu et al. 2014). A variety of additives have been used to enhance the mechanical properties of paper, textile, wood, and packaging. Because of its unique mechanical strength, surface qualities, accessibility, adaptable hydrophilicity, quantity of raw materials, and inexpensive synthesis process, carboxymethyl cellulose (CMC) is the most promising cellulose derivative (Farooq et al. 2020; Seddiqi et al. 2021; Fernández-Santos et al. 2022). The hydrophilic character, good film-forming abilities, high viscosity, and adhesive performance facilitate its application. Biomedical engineering, food, paper, textile, pharmaceutical industries, wastewater treatment, and energy production are a few examples of these sophisticated application sectors (Kukrety et al. 2018; Rahman et al. 2021).

The compatibility of titanium dioxide-based nanoparticles (TiO_2_NPs) with living cells has been studied in numerous papers. To track how NPs infiltrate living creatures’ cells, the interaction of TiO_2_NPs and Chinese hamster ovary (CHO) cells was monitored. It was established that the interaction of TiO_2_NPs with the cell’s plasma membrane was what caused the increased maximal depth of the pits following the exposure of TiO_2_NPs (Batiuskaite et al. 2022). After incubation at 37 °C, the morphological alterations and stimulation of aggregation and fibrillation of β-amyloid fragment 1–40 (βA) and α-synuclein protein attributed to the interaction of TiO_2_NPs and zinc oxide nanoparticles (ZnONPs) with amyloid proteins (Slekiene et al. 2022).

On some artifacts, a commercially available derivative form called Phytagel plant cell (PGP) has been applied (Domon Beuret et al. 2014). Its selection was influenced by its adherence to vertical surfaces, ease of removal, and compatibility with the chosen active agents (i.e., microbes). The gelling properties of this material may be observed at low concentrations (0.5 to 5 g/L) and across a wide pH range, in addition to its transparency, which is viewed as an advantage because it makes the treatment easier to observe and track (Domon Beuret et al. 2014). PGP can be used for enhancing the properties of CMC while film formation. As known, PGP is fabricated from a bacterial substrate that is composed of glucuronic acid, rhamnose, and glucose (Jacques et al. 2020). For instance, the bacterium *Sphingomonas elodea* produces the polysaccharide gellan gum (Wu et al. 2011; Prajapati et al. 2013). Similar to agar, it acts best when applied warmly and, once created, forms a hard gel that can be impregnated with both acidic and basic liquids (Guilminot et al. 2019). Due to the gelling property of PGP plates, it increases the hairiness in plants and controls the mechanical strength (Buer et al. 2000).

TiO_2_NPs are an inorganic nanoparticle that has been employed in a variety of applications including textiles, polymers, cosmetics, and food packaging and alleviate the heavy metal toxicity (Mallakpour and Jarang 2018; Youssef and El-Sayed 2018; Mohr et al. 2019; Liao et al. 2020; Abutalib and Rajeh 2021; Kumar et al. 2023). As a result, environmentally acceptable methods for producing TiO_2_NPs on a bigger scale with less toxicity have been developed (Baranwal et al. 2018).

In order to clean or shield manuscripts or other organic materials from contamination, microbial development, or even heavy metals from soil, it is urgently encouraged to adopt unique eco-friendly biofilms (Bradu et al. 2022; Chaturvedi et al. 2006, 2007; Habeche et al. 2023; Misra et al. 2009; Natarajan et al. 2020; Shen et al. 2021). For instance, biofilms composed of surface-attached bacteria embedded in an extracellular polymeric substance matrix demonstrated their effectiveness against *Staphylococcus aureus*, *S. epidermidis*, *Escherichia coli*, *Klebsiella pneumoniae*, *Pseudomonas aeruginosa*, and *Enterococcus faecalis* (Weldrick et al. 2019). The promoted biofilm formation from chitosan with increasing adhesion of the microstructured surfaces led to increased antibacterial action (Estevam-Alves et al. 2016).

Herein, the current work was designed to prepare an eco-friendly biofilm comprising two environmental polymers: CMC and PGP. To increase the film efficiency, the prepared TiO_2_NPs with three volumes were added to the biofilm solutions. CMC/PGP biofilms loaded with the three volumes of TiO_2_NPs were fully investigated in terms of particle shape, average hydrodynamic size, stability, morphological features, swelling (%), and mechanical properties. The work was extended to evaluate the antimicrobial biofilms for possibly protecting the old manuscripts from microbial growth.

## Materials and methods

### Chemicals

Titanium isopropoxide (TTIP, 99%) was purchased from Sigma-Aldrich Co. (USA). Nitric acid was purchased from Merck Co. (Germany). Carboxymethyl cellulose (CMC) was purchased from Across Co. (Germany). Meanwhile, Phytagel plant cell (PGP) was purchased from B&V Laboratory Chemicals (Italy). Glycerol and epichlorohydrin were purchased from WIN lab Co. (India). All other chemicals were used as received without purification.

### Preparation of titanium dioxide nanoparticles (TiO_2_NPs) and the biofilms based on CMC, PGP, and TiO_2_NPs

The sol–gel method was used to synthesize TiO2NPs by dispersing 7 mL of TTIP in 80 mL of distilled water (DW). Nitric acid was then used to further treat the mixture until the pH reached 1.9. After 36 h on a magnetic stirrer, the solution was aged for 6 h at 40 °C, yielding TiO_2_NP sol. The suspensions were vacuum-dried at room temperature to produce the powdered TiO_2_NPs. The powder was then dried at room temperature after being washed three times with DW to get rid of the unreacted components. Subsequently, 0.5 g of the dried TiO_2_NPs was suspended in 30 mL of DW under vigorous stirring.

To produce the biofilms based on CMC, PGP, and TiO2NPs, a viscous solution of CMC was prepared by dissolving 10 g in 300 mL of DW and keeping the mixture at room temperature under mechanical agitation until the CMC was completely dissolved. Secondly, a PGP solution was made by dissolving 10 g of PGP in 300 mL of DW and stirring magnetically at 70 °C for 30 min.

The two aforementioned solutions were then combined with various volumes of the synthesized TiO_2_NPs, as shown in Table 1. Mechanical homogenization was used to homogenize the resulting mixtures of CMC, PGP, and TiO_2_NPs, which also lacked any observable precipitations or phase separations.

**Table 1:** Volumes of the ingredients used in the manufacture of each biofilm formation

Film code	CMC volume (mL)	PGP volume (mL)	TiO_2_NPs (mL)	Glycerol concentration (mL)	Epichlorohydrin (mL)	Total volume (mL)
CMC/PGP	36.5	40	Zero	2.5	1	80
CMC/PGP/TiO_2_-1	35.5	40	1	2.5	1	80
CMC/PGP/TiO_2_-2	34.5	40	2	2.5	1	80
CMC/PGP/TiO_2_-3	32.5	40	4	2.5	1	80

Glycerol was added after complete homogeneity to enhance the plasticizer’s properties for the film-forming polymers. Following that, a constant amount of the crosslinking agent epichlorohydrin was added to the aforementioned solutions, which were then mechanically agitated for 20 min. Once the solution bubbles had been removed and it had become clear, the generated solution was ultrasonically processed before being cast into films on 9 cm × 13 cm plates. After that, the plates were then dried in an oven with forced air at 35 °C. The biofilms were placed in a temperature and humidity-controlled chamber (25 °C and 50% RH) for at least 60 h after being removed from the dishes before being put through mechanical testing.

### Characterization of the prepared TiO_2_NPs and the produced biofilms

The particle shape of the produced TiO_2_NPs was examined using a transmission electron microscope (TEM, JEOL, Japan) at three different magnifications. Diluted TiO_2_NP solution was applied on carbon-coated copper grids, and the grids were then allowed to air dry. Dynamic light scattering (DLS) was used to examine the average hydrodynamic particle size and particle stability (zeta potential).

The surface structure of the produced films was examined using scanning electron microscopy (SEM, TESCAN, Czech Republic) with an accelerating voltage of 20 kV. Meanwhile, the elemental analysis for all prepared films was assessed via energy-dispersive X-ray that connected with field emission scanning electron microscopy (FESEM-EDX, Quanta FEG 250, Czech Republic).

The produced films were tested for tensile strength (MPa), elongation at break (%), and tear strength (g) using an Instron Universal testing machine no. 4301 (Standard Test Method for Tensile Properties of Plastics, Designation D638-96) with a 50 mm/min extension rate. The Instron Universal testing machine no. 4301 was used to assess the tear strength of the films. The test was performed in accordance with American Society for Testing and Materials (ASTM D1938). The sample was 120 mm long and 25 mm wide, with a 50 mm incision in the middle of one end.

The prepared biofilms’ swelling qualities were also determined in the following way: the film was cut to a certain size and its original weight was weighed. It was soaked in 30 mL of distilled water for 30 min at room temperature. The wet weight of the swollen biofilm was weighed after it was dried. Immersion and weighing were performed several times until the weight was stable.

$$\mathrm{Swelling }\left(\mathrm{\%}\right)=(\frac{(X1-X0)}{X0}) \times 100$$

*X*1 is the wet weight of the film after immersion, and *X*0 is the dry weight of the film before immersion. The studies were carried out at a speed of 50 mm/min.

### Culturing and molecular identification of the isolated microorganisms

Cultures from a manuscript found in the storage room at the library of Cairo University, Egypt, were swabbed and taken. Potato Dextrose Agar (PDA) (Difco, Inc., Detroit, MI, USA) plates were used to culture the mold found in the swabbed samples. The fungal plates were then incubated at 27 ± 2 °C for 7 days. After that, the obtained fungus was purified and maintained in slant tubes at 4 °C in the fridge. Meanwhile, to isolate the bacterial load cultures, the swapped sample was cultured in nutrient broth (NB) medium at 30 °C for 72 h with shaking at 180 rpm. Then, 1 mL of the incubated broth culture was streaked on nutrient agar plates. The obtained bacterium culture was resuspended in NB, and the resultant bacterial suspension (5 mL) was span down at 6000 × *g* for 10 min at 4 °C to collect the bacterial pellet which was reserved to further molecular analysis (Tan et al. 2019).

To amplify the target gene or region from the obtained fungus or the bacterium, the reaction temperature is increased to 95 °C and the reaction is incubated for 5 min and then cycles of heating at 95 °C for 30 s, annealing at the specific temperature for each primer set used in Table 2 and extension at 72 °C for 30 s, and the reaction was ended after 7 min at 72 °C. The amplification reaction was performed by Go Taq flexi (Promega, USA, Cat# M8305) and was composed of 5 μL Go Taq buffer (5X), 2 μL (25 mM MgCl_2_), 2 μL (2.5 mM dNTPs), 2.5 μL (10 pmol primer set), and 1.25 unit Go Taq polymerase (On et al. 2013).

**Table 2:** Primers used for sequencing to identify the isolated microorganisms

Identity of the microorganism	Target gene/region	Primer name	Primer sequence (5′-3′)	Annealing temperatures
Fungus	ITS region	ITSF	CTT GGT CAT TTA GAG GAA GTA A	55 °C
		ITSR	TCC TCC GCT TAT TGA TATGC	
Bacterium	16S ribosomal RNA gene	16S8FWD	AGAGTTTGATCCTGGCTCAG	50 °C

The amplified fragments of the two organisms were purified and subjected to sequence analysis using the Big TriDye sequencing kit (ABI Applied Biosystems) by the facility of Macrogen Co., at Seoul, Korea (Fig. S1 and Fig. S2). The obtained sequences were then deposited in the GenBank portal in order to obtain their accession numbers.

### Colonization test

The cotton paper samples were prepared from pure cotton in the Egyptian National Library and Archives. The test samples were cut into small pieces (20 × 20 mm) using the scalpel for the colonization test. The thermal aging was conducted between the temperatures of 80 °C and 65% relative humidity for 240 h during 10 days, which is equivalent to 50 years under normal aging conditions (ISO-5630–3,1996), in the National Measurements and Calibration Center, Egypt. After that, the produced cotton papers were autoclaved at 121 °C in an oven at 105 °C for 24 h. Sterilized distilled water (10 mL) was added to culture plates containing PDA medium to prepare the spore suspension of the fungus (7 days old), and then, these spores were spread using a camel hairbrush. Then, the infected paper was placed on the glass slide in a sterilized Petri dish. For the bacterium colonization, the cell density was adjusted to the required bacterial density [1 × 10^8^ colony forming units (CFU/mL)] (Chen et al. 2014), where fresh 1 mL of the prepared bacterial suspension was used for coating the cotton paper.

The colonization process was kept in controlled growth chambers for 6 months, and then, the inoculated cotton papers with the microorganisms were evaluated and compared with the standard samples (un-inoculated).

### Antimicrobial activity by eco-friendly films

#### Biofilm setup

The created biofilms with TiO_2_NPs were first activated by UV light, which was applied using a UV lamp with a working power of 15 W and a wavelength of 364 nm for 10 h. The UV source was 10 cm away from the prepared biofilms loaded with TiO_2_NPs (El-Hossary et al. 2020).

**Fig. 1:**
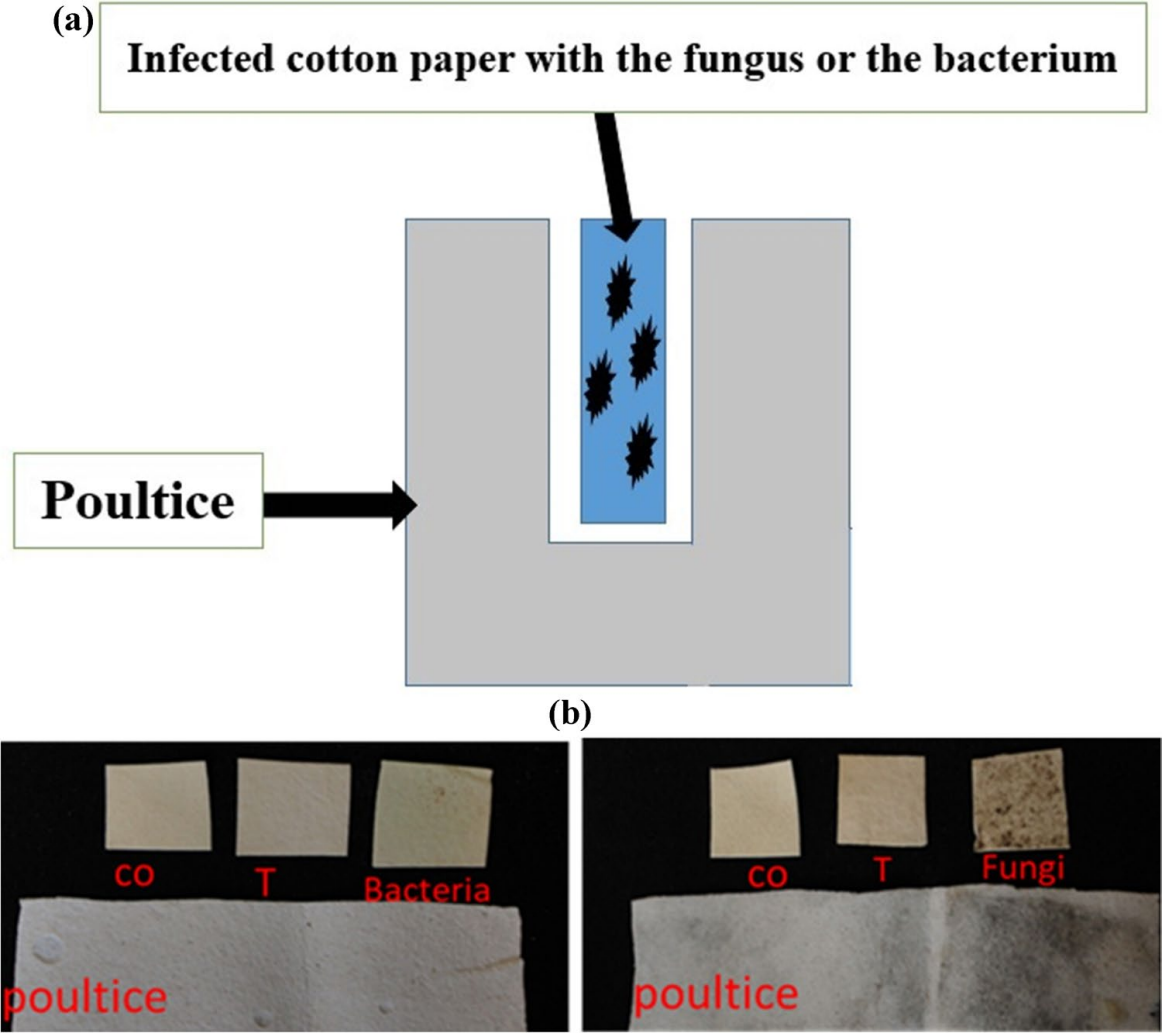
Antimicrobial activity of the prepared biofilms against the microorganism-infected cotton paper. **a** A biofilm poultice in the form of a sandwich. **b** The observation process of cleaning for cotton paper after being treated with the biofilms. CO: control; T: treated

The infected cotton paper was sandwiched between the prepared biofilm folds to test the prepared biofilms’ antimicrobial activity against the isolated microorganisms (Fig. 1a). Figure 1b displays the visual observations of the cleaning process for cotton paper after being treated with the biofilms. The treated paper was left at room temperature 30 °C beside the window to be directly exposed to natural sunlight for 2 h in March between 8 am and 10 pm to assess field efficiency and 70% RH.

#### Exposure conditions

The biofilm setup was left at room temperature (30 °C) beside the window to be directly exposed to natural sunlight (the continuous energy source for the activation of TiO_2_) (Liao et al. 2020; Quiñonez et al. 2020) for 2 h in March between 8 am and 10 pm to assess field efficiency and 70% RH. Tests were undertaken on triplicates.

#### SEM evaluation

The infected, treated, and standard (control) samples of cotton paper were evaluated using scanning electron microscope analysis (SEM, TESCAN, Czech Republic) at an accelerating voltage of 20 kV.

#### Statistical analysis

Data of the mechanical properties [tensile strength (MPa), elongation at break (%), and tear strength (g)] and the thickness (mm) and swelling (%) of the prepared films were statistically analyzed using one-way analysis of variance. The Least Significant Difference (LSD) was used to measure the difference between means at a 0.05 level of probability.

## Result and discussion

### Characterization of TiO2NPs

**Fig. 2:**
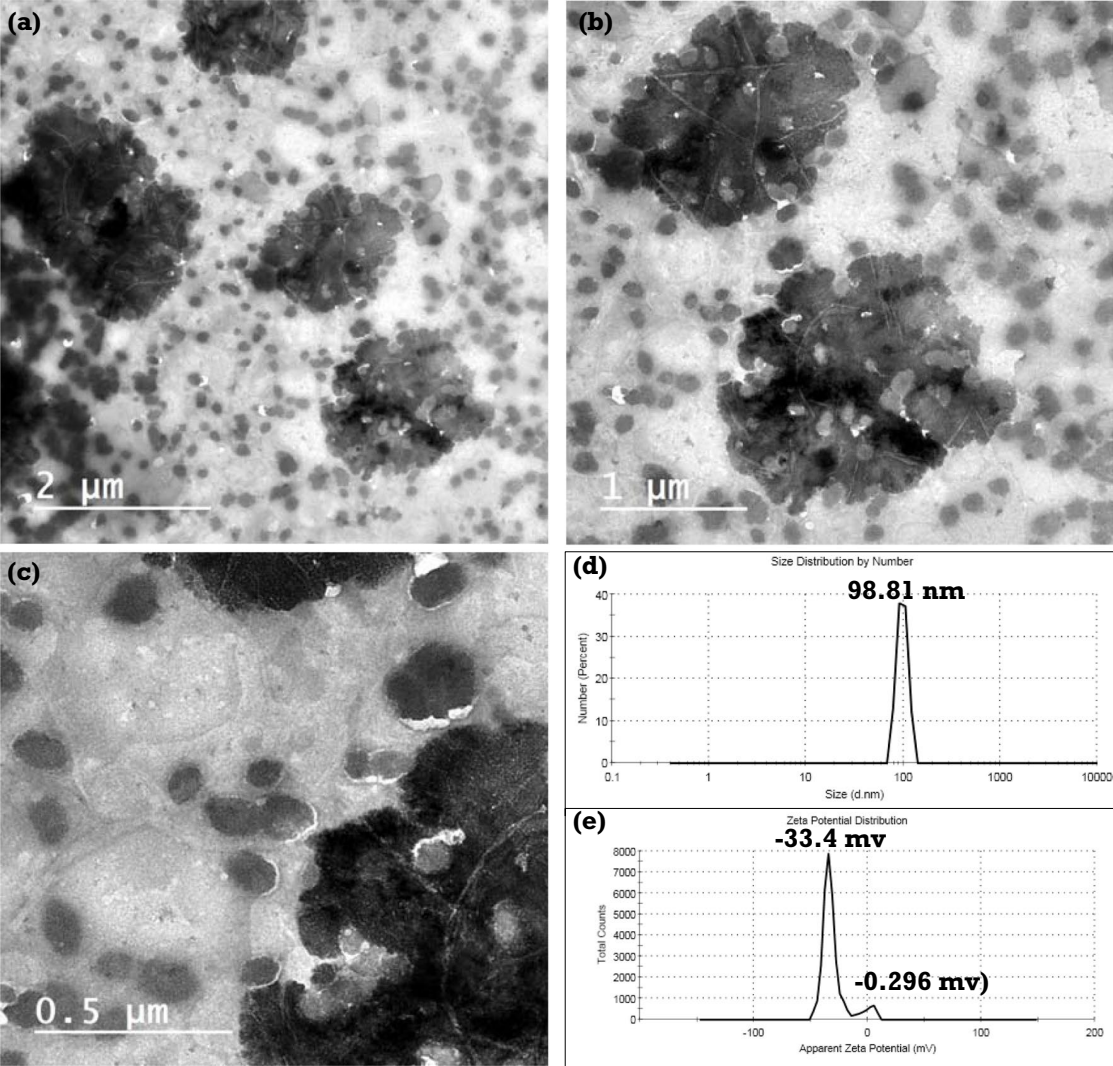
TEM at three different magnifications (**a**, **b**, **c**), average particle size (**d**), and zeta potential (**e**) of the synthesized TiO_2_NPs

The particle shape of titanium dioxide nanoparticles (TiO_2_NPs) was investigated using a transmission electron microscope (TEM). The sample was studied at three different magnifications, as shown in Fig. 2a–c, and it was found that the size of TiO_2_NPs has a fairly distinct form and good distribution. Additionally, a portion of these spherical particles was clustered and turned aggregated particles.

As shown in Fig. 2d, the average hydrodynamic size determined by dynamic light scattering (DLS) indicates that the percentage of generated TiO_2_NPs is close to 98.81 nm. Additionally, a major peak at − 33.4 mV is seen in the zeta potential of the synthesized TiO_2_NPs (Fig. 2e), indicating that the TiO_2_NPs are negatively charged and exhibit excellent stability against agglomeration (Ji et al. 2010). The formation of TiO_2_NPs with zeta potential exceeding + 30 and − 30 mV demonstrates that TiO_2_NPs (− 33.4 mV) were effectively prepared (Cakmak et al. 2020).

### Surface structure of biofilms

**Fig. 3:**
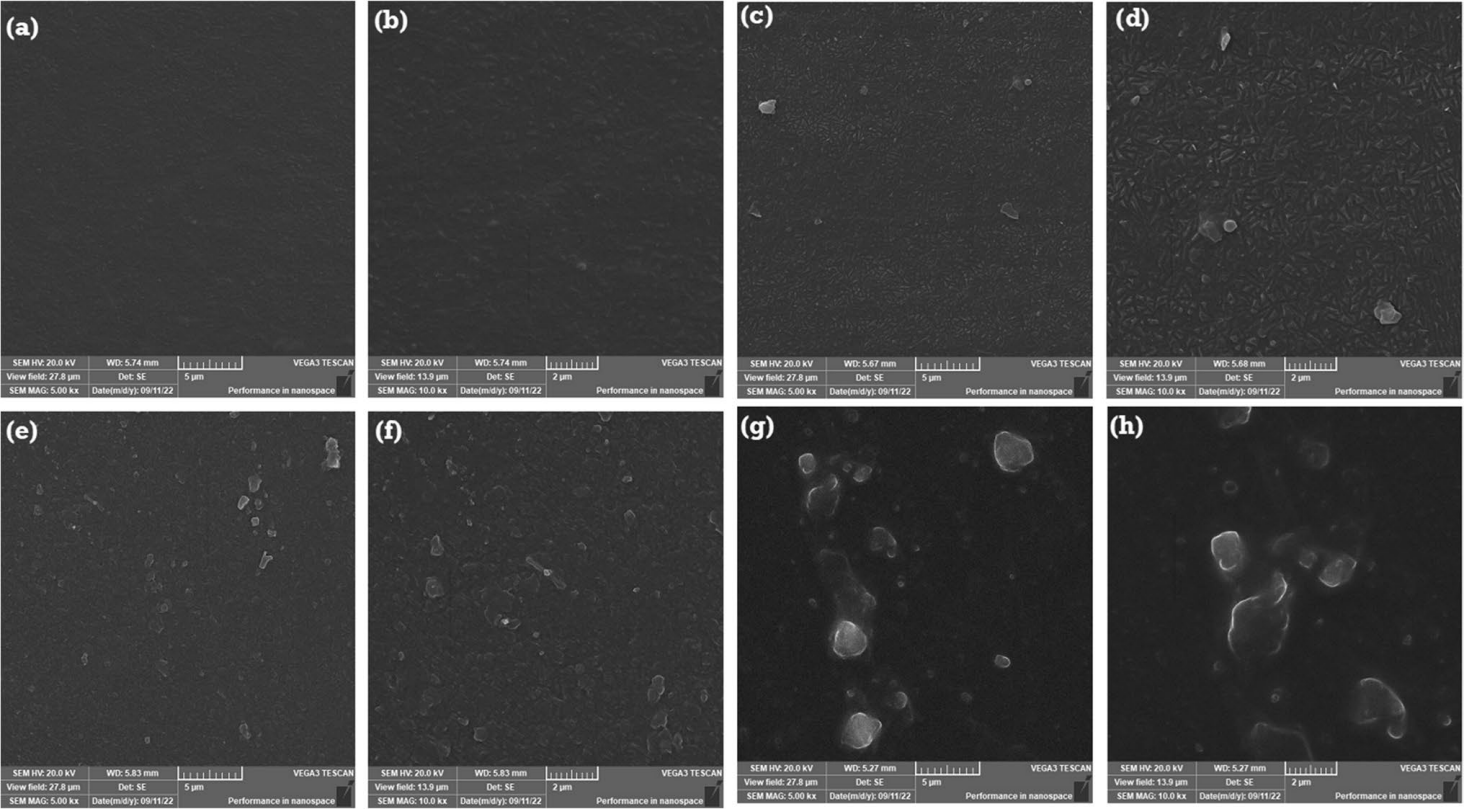
Field emission scanning electron microscopy images of **a**, **b** CMC/PGP, **c**, **d** CMC/PGP/TiO_2_-1, **e**, **f** CMC/PGP/TiO_2_-2, and **g**, **h** CMC/PGP/TiO_2_-3 of the synthesized TiO_2_NPs

Regarding the surface structure of the produced biofilms, as depicted in Fig. 3, it can be seen that the biofilm based on carboxymethyl cellulose/Phytagel plant cell (CMC/PGP) has a smooth, spotless, and continuous surface without any signs of cracking, the development of porous structures, or the deposition of any visible particles (Fig. 3a, b). The surfaces of the biofilms made from CMC and PGP and mixed with various volumes of TiO_2_NPs, however, are entirely different. The biofilm (CMC/PGP/TiO_2_-1) formed with the low concentration of TiO_2_NPs has a rough structure and impressive deposition for small spherical TiO_2_NPs, as illustrated in Fig. 3c, d.

The formation of the biofilms CMC/PGP/TiO_2_-2 and CMC/PGP/TiO_2_-3 with varied surface appearances is caused by an increase in the volume of TiO_2_NPs. The film surface was found to have some agglomerates, some of which may be TiO_2_NPs, as illustrated in Fig. 3e–h. The lack of separation between the TiO_2_NPs and biofilm-forming polymer phases suggests that all three of these components have a consistent morphology and get along well with one another. The hydrogen bonding interactions and chemical resemblances between the filler and matrix are presumably what caused the homogeneity to be seen.

**Fig. 4:**
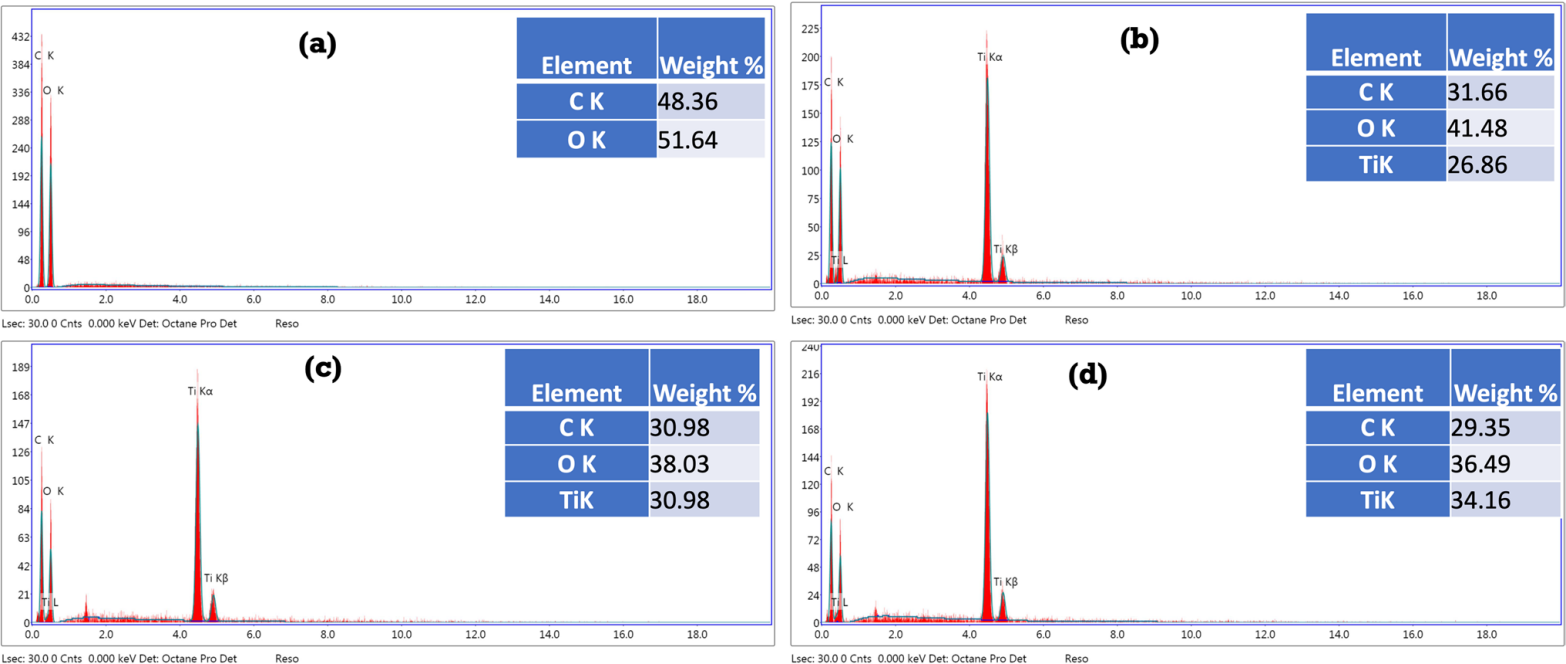
Elemental analysis for the prepared biofilms with and without TiO_2_NPs: **a** CMC/PGP, **b** CMC/PGP/TiO_2_-1, **c** CMC/PGP/TiO_2_-2, and **d** CMC/PGP/TiO_2_-3

Energy-dispersive X-ray (EDX) was used to confirm the components that were used to produce the biofilms. The elemental analysis of CMC/PGP, CMC/PGP/TiO_2_-1, CMC/PGP/TiO_2_-2, and CMC/PGP/TiO_2_-3 is shown in Fig. 4a–d. Carbon and oxygen were the only two elements found in the CMC/PGP biofilm (Fig. 4a). The biofilms made from CMC/PGP/TiO_2_-1, CMC/PGP/TiO_2_-2, and CMC/PGP/TiO_2_-3, however, also include titanium (Ti) in addition to carbon and oxygen (Fig. 4b–d), demonstrating the presence of a TiO_2_NP-loaded CMC/PGP biofilm.

The percentage weight for each element is displayed in the inset tables. It was noted that compared to the other produced biofilms, the Ti content was higher in CMC/PGP/TiO_2_-3. The elemental analysis phenomenon confirmed that the two film components and TiO_2_NPs are compatible.

### Mechanical properties of the biofilms

**Fig. 5:**
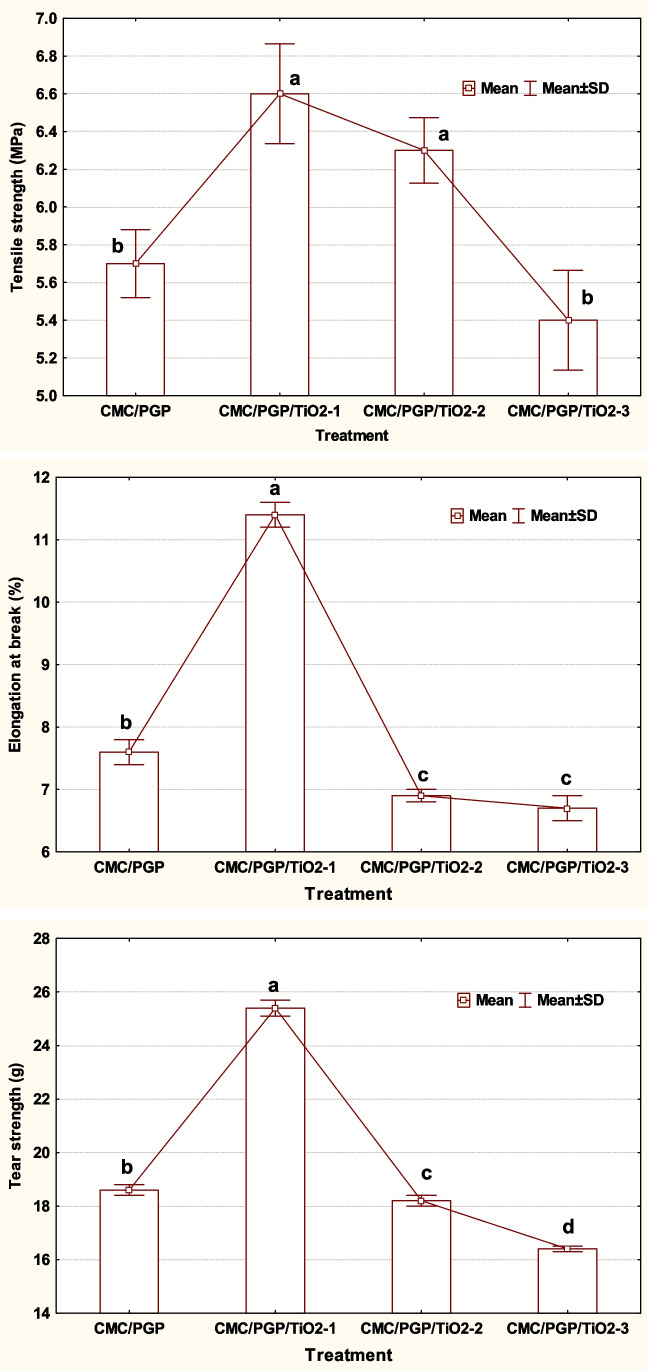
Tensile strength (MPa), elongation at break (%), and tear strength (g) of the prepared films. According to LSD at 0.05 level of probability, means with the same letter for the same characteristic are not statistically different

Figure 5 displays the formed biofilms based on CMC/PGP, CMC/PGP/TiO_2_-1, CMC/PGP/TiO_2_-2, and CMC/PGP/TiO_2_-3 in terms of their tensile strength (MPa), elongation at break (%), and tear strength (g). The mechanical properties of the manufactured biofilms are often attributed to the interaction of CMC, PGP, glycerol, and TiO_2_NPs. The created biofilms’ tensile strengths were discovered to be 5.7, 6.6, 6.3, and 5.4 MPa, respectively.

As evidenced by the two biofilms CMC/PGP/TiO_2_-2 and CMC/PGP/TiO_2_-3, the tensile strength of the blends decreased as the volume of TiO_2_NPs increased. The mechanical properties with the greatest values were found to be in the CMC/PGP/TiO_2_-1 biofilm (Dash et al. 2019; Fathi et al. 2019; Dong et al. 2021). The tensile strength of the resulting biofilms decreases as the volume of TiO_2_NPs rises. A good correlation was found between the elongation of the biofilms at breaking (Fig. 5) and the tensile strength result, which was 7.6, 11.4, 6.9, and 6.7%, respectively.

The mechanical properties of the produced films are improved by blending with a small amount of TiO_2_NPs, which is connected to hydrogen bond interaction in CMC and PGP. The tear strength value (g) was increased by adding a small amount of TiO_2_NPs, as found in the biofilm CMC/PGP/TiO_2_-1 (25.4 g). This value decreased while increasing the volume of TiO_2_NPs in the blended films CMC/PGP/TiO_2_-2 (18.2 g) and CMC/PGP/TiO_2_-3 (16.4 g) compared to CMC/PGP (18.6 g). Overall, it was found that there was little difference in the blended biofilms’ values for tensile strength, elongation at break (%), and tear strength.

As shown in Table 3, the results of measurement of biofilm thickness consists of CMC/PGP, CMC/PGP/TiO_2_-1, CMC/PGP/TiO_2_-2, and CMC/PGP/TiO_2_-3 were 0.176, 0.313, 0.213, and 0.333 mm. When put into the mold, homogeneity properties may have contributed to the four prepared films’ inconsistent thickness, as was seen. The total solids in each portion were not equal as a result, and the drying time varied.

**Table 3:** Values of thickness (mm) and swelling (%) of the prepared biofilms

Sample	Thickness (mm)	Swelling (%)
CMC/PGP	0.176d ± 0.034*	422a ± 5.3
CMC/PGP/TiO_2_-1	0.313b ± 0.064	407b ± 6.6
CMC/PGP/TiO_2_-2	0.213c ± 0.073	333c ± 4.5
CMC/PGP/TiO_2_-3	0.333a ± 0.012	216d ± 2.4

*Values are mean ± SD. Means with the same letter within the same column are not significantly different at 0.05 level of probability

The cut films loaded with various volumes of TiO_2_NPs (CMC/PGP, CMC/PGP/TiO_2_-1, CMC/PGP/TiO_2_-2, and CMC/PGP/TiO_2_-3) were submerged in a Petri dish containing 30 mL of distilled water to test the films’ water resistance. The obtained results (Table 3) showed that the swelling (%) reduces as the proportion of TiO_2_NP-loaded CMC/PGP biofilm increases. This is because the biofilm absorbs less water. The action of TiO_2_NPs, which are able to form intermolecular hydrogen bonds and coordination bonds with the hydroxyl groups of biopolymer films, may be responsible for the decrease in swellability (%) by increasing the films’ resistance to water (Hou et al. 2019).

### Growth inhibition of microorganisms

#### Isolated microorganisms

From a manuscript found in the storage room in the library of Cairo University, the fungus *Aspergillus sydowii* was isolated, identified, and deposited under the accession number MG991624 with exhibited similarity of 98.86% (Fig. S3). Additionally, a novel bacterial strain, designated uncultured bacterium, was isolated and exhibited a similarity of 99.53% to members of other genera in the family Nevskiaceae. *Nevskia terrae* with accession number AB806800 (Fig. S4) is Gram-negative and strictly aerobic and formed translucent white-colored colonies from the genus of *Nevskia*. *N. terrae* was isolated from soil in Korea (Kim et al. 2011).

#### Growth of A. sydowii on cotton paper

**Fig. 6:**
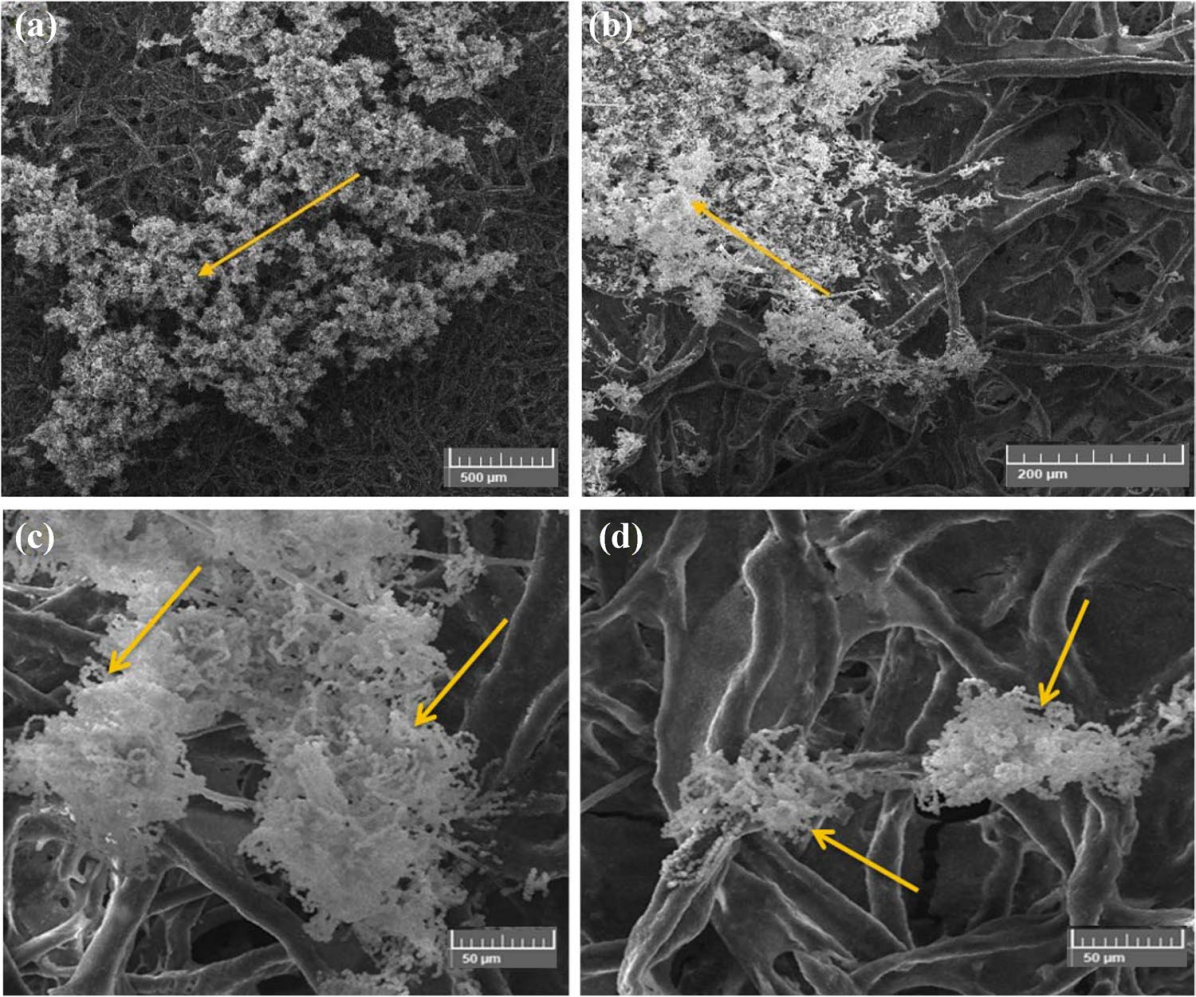
SEM images of *Aspergillus sydowii*’s growth patterns on cotton paper. The images highlight distinctive characteristics such as dense mycelium, mass branching spore/conidia chains, conidiophores, and hyphae concentrated on fiber paper (**a** at bar 500 μm, **b **at bar 200 μm); morphological features of conidia head with a vesicle characteristic (**c **at bar 50 μm); and conidiophore structure with metulae and globose conidia (**d** at bar 50 μm). The direction of *A. sydowii*’s development is shown by arrows

The fungus shown in SEM images (Fig. 6) was highly profuse in the colonized sample (Fig. 6a, b). The growth was typically *A. sydowii* due to their globose to sub-globose morphology characteristic (Fig. 6c, d). The microstructure of *A. sydowii* was studied using SEM by high magnification. *A. sydowii* cells were grown on the surface and were dense porous on the cotton paper after incubation for 6 months with *A. sydowii* due to their globose to sub-globose morphology characteristic (Soler-Hurtado et al. 2016). Dense mycelium growth and well-developed mass branching adhered to cellulose were observed (Ganesh Kumar et al. 2021). However, with the fungal attack, there was a clear spread of *A. sydowii*, causing structural changes in the paper.

#### Growth inhibition of A. sydowii on cotton paper treated with the biofilms

**Fig. 7:**
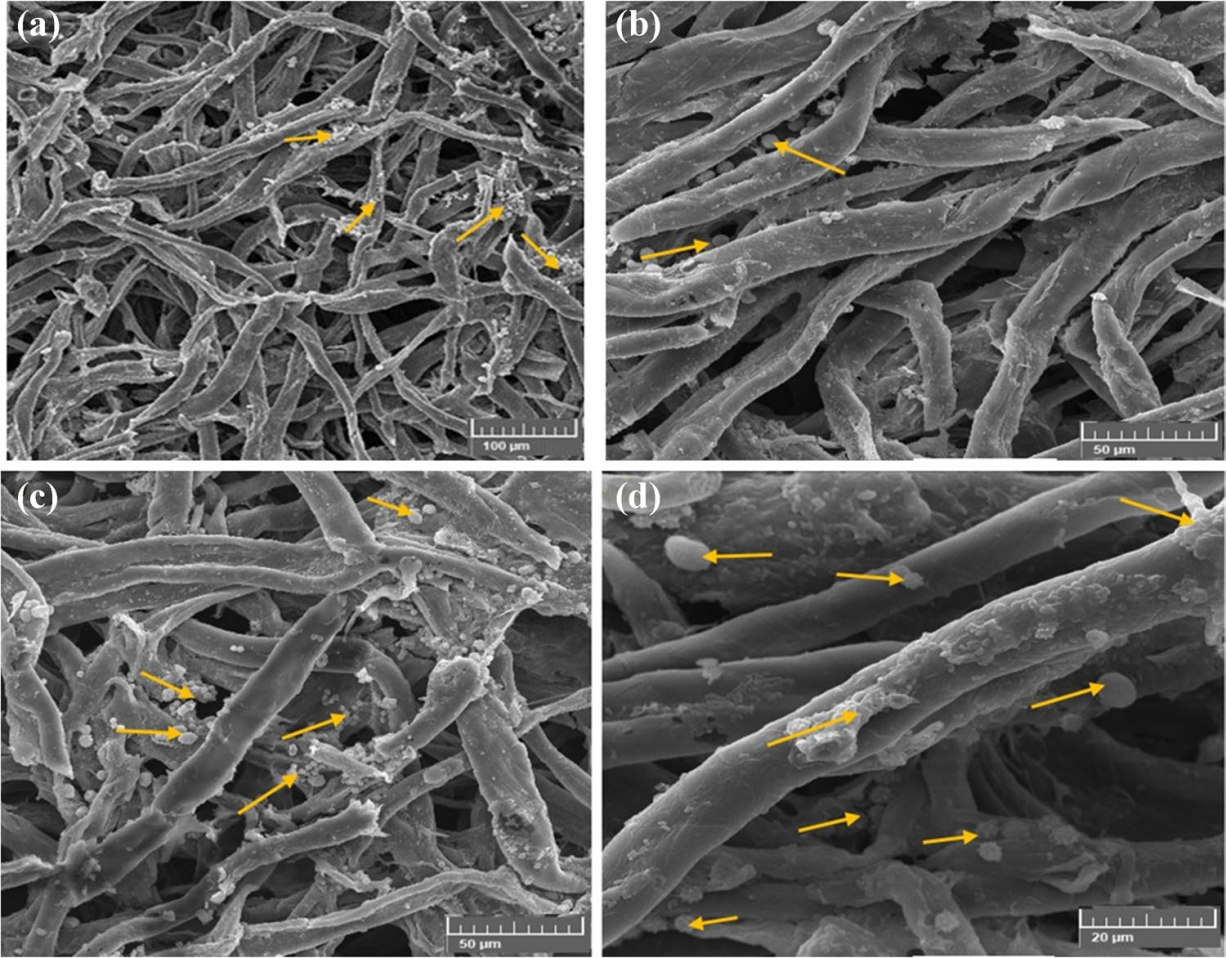
Inhibition patterns of *A. sydowii* when the cotton paper was treated with CMC/PGP. The growth development of *A. sydowii* is shown by arrows. Images were taken at bar 100 μm (**a**), bar 50 μm (**b**), bar 50 μm (**c**), and bar 200 μm (**d**)

*A. sydowii* was significantly impacted by the poultice’s CMC/PGP formulation composition (Fig. 7a). The hyphae and spores of *A. sydowii* were absorbed when the poultice was given to the infected cotton paper, which resulted in the cleansing of the cotton fibers but had an impact on the fibers when seen under a scanning electron microscope, where some cracks were seen (Fig. 7b, c). Because it was wet, the poultice absorbed the fungus growths, which led to the cleaning of the treated samples from the fungal growths. The growth was slowed after 6 months of incubation, and the spore’s appearance and shape changed. With limited conidia and tiny hyphae inhibited between the fibers of *A. sydowii*, the hyphae were torn to bits and weakened, making the fiber apparent (Fig. 7d).

**Fig. 8:**
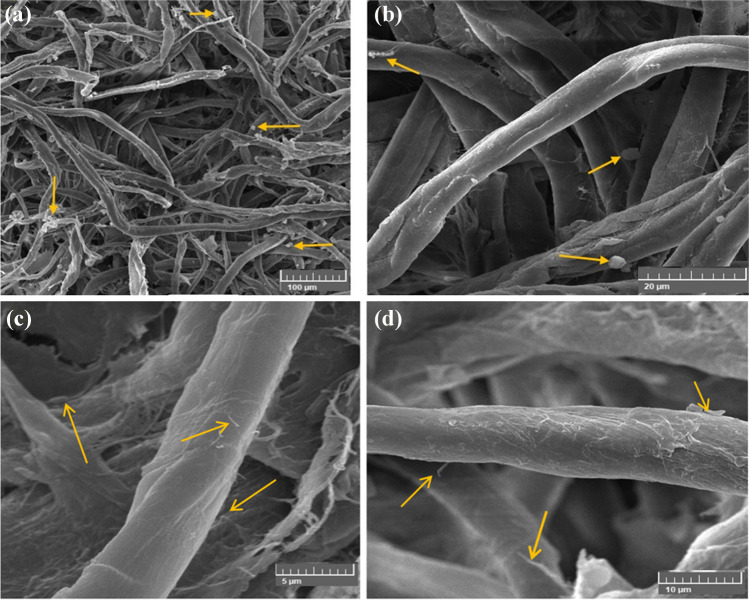
Inhibition patterns of *A. sydowii* when the cotton paper was treated with CMC/PGP/TiO_2_-1. The growth development of *A. sydowii* is shown by arrows. Images were taken at bar 100 μm (**a**), bar 20 μm (**b**), bar 5 μm (**c**), and bar 10 μm (**d**)

The cotton fibers infected with *A. sydowii* and treated with a CMC/PGP/TiO_2_-1 poultice are shown in Fig. 8. The growth of *A. sydowii* was extremely slow, and the spore production was inhibited (Fig. 8a, b). *A. sydowii*’s tiny hyphae that inhibited between the fibers had few conidia, and they were broken to pieces and weakened, which made the fiber visible. Although there were minor cracks in the cotton fibers, the poultice prevented the growth of the fungus (Fig. 8c, d). The fungal growths were absorbed by the poultice that developed on the cotton paper, which allowed the treated samples to be free of the fungal growth.

**Fig. 9:**
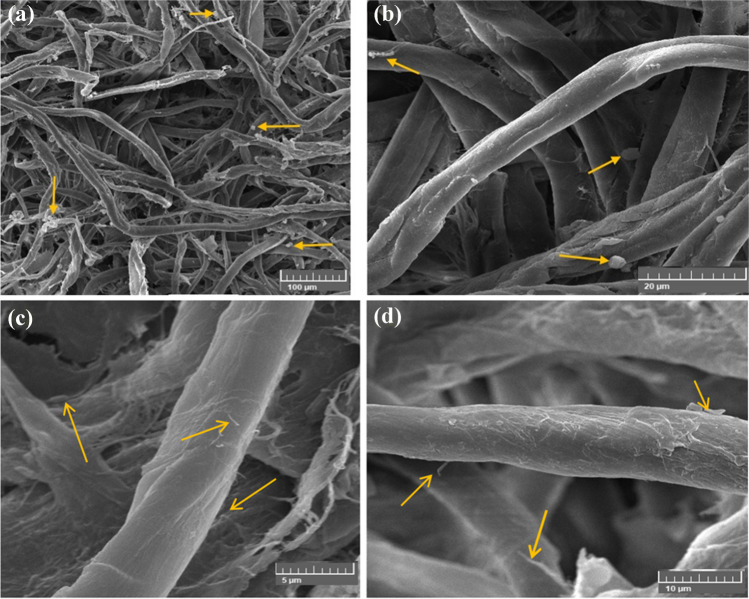
Inhibition patterns of *A. sydowii* when the cotton paper was treated with CMC/PGP/TiO_2_-2. The growth development of *A. sydowii* is shown by arrows. Images were taken at bar 100 μm (**a**), bar 20 μm (**b**), bar 5 μm (**c**), and bar 10 μm (**d**)

The fungus’ growth was prevented by the poultice composition of CMC/PGP/TiO_2_-2, which also demonstrated the removal of the fungus’s growth from the fibers (Fig. 9a, b). It also resulted in the preservation of the fibers by covering them with the material loaded on the poultice (Fig. 9c, d), proving that it is one of the best poultices utilized in terms of controlling the fungus and protecting the fibers. The treated cotton paper was cleaned since the poultice removed the fungus growths.

**Fig. 10:**
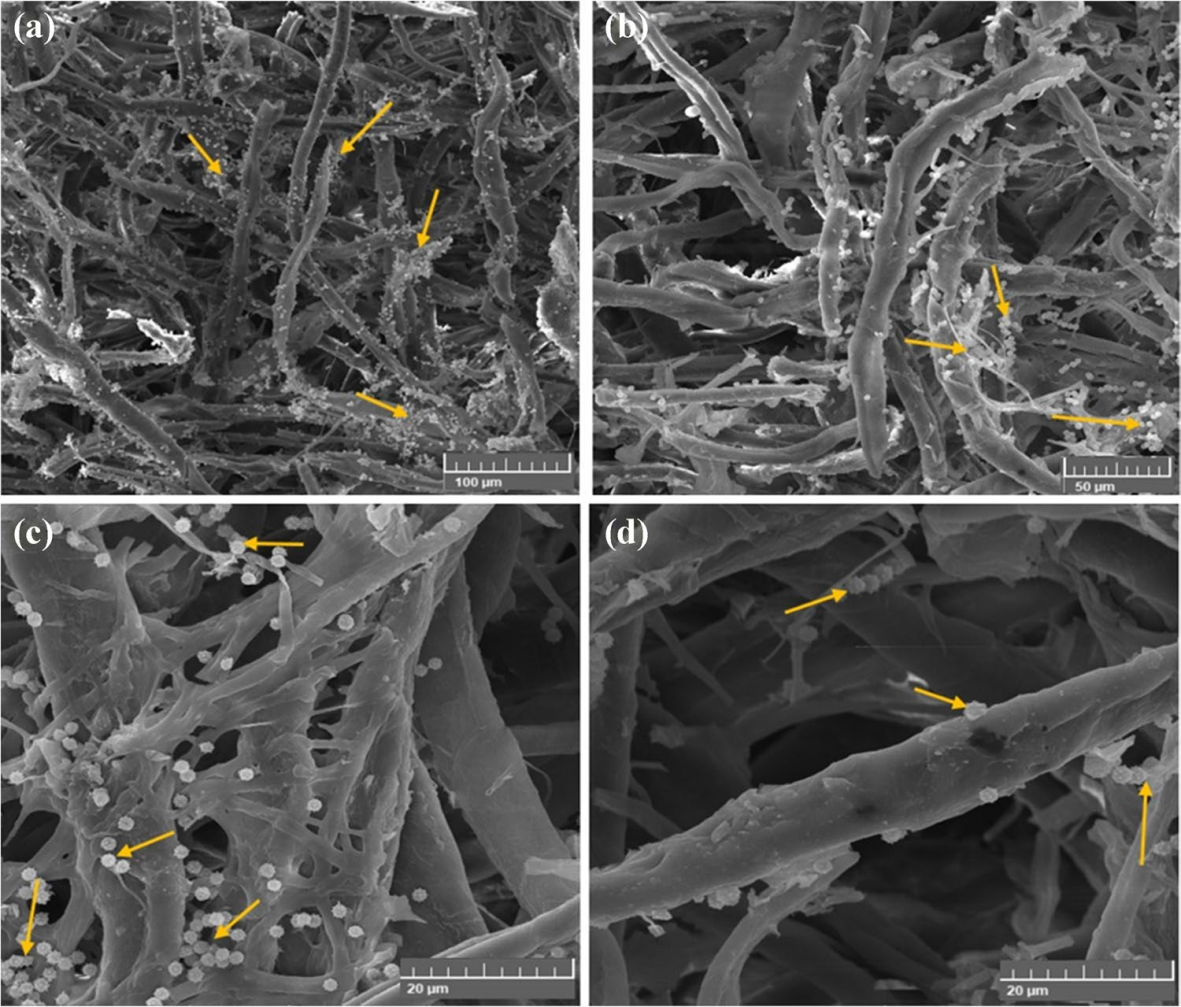
Inhibition patterns of *A. sydowii* when the cotton paper was treated with CMC/PGP/TiO_2_-3. The growth development of *A. sydowii* is shown by arrows. Images were taken at bar 100 μm (**a**), bar 50 μm (**b**), and bar 20 μm (**c** and **d**)

The fungal germs are propagated on the fibers despite being separated from the conidia (Fig. 10a, b), which are shown to be widely dispersed across and between cotton fibers (Fig. 10b, c). These results suggested that the fungus was only marginally affected by the CMC/PGP/TiO_2_-3 poultice.

### Growth of Nevskia terrae on cotton paper

**Fig. 11:**
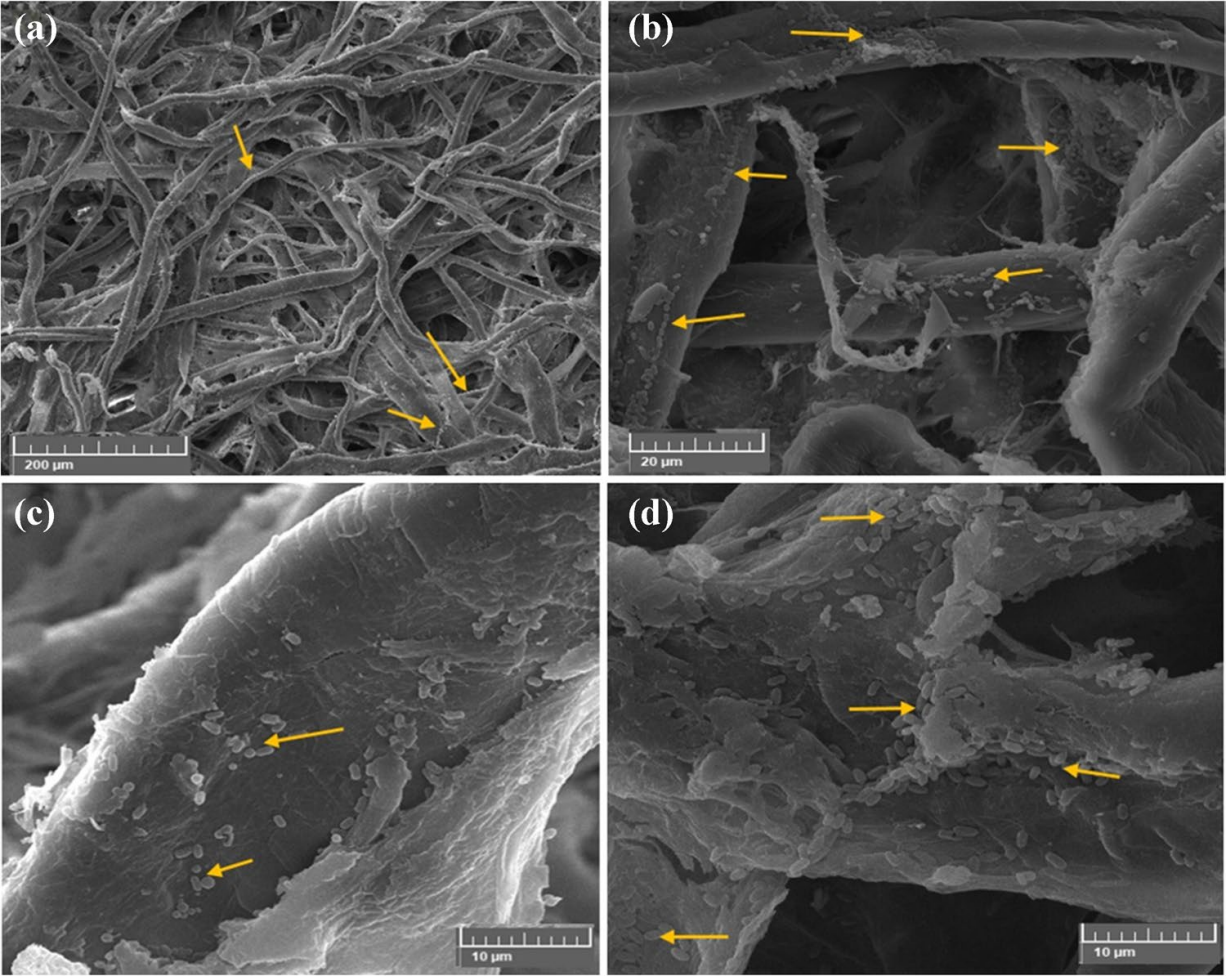
SEM images of cotton paper samples inoculated by *Nevskia terrae* for 6 months showing bacillus shape. The growth development of *N. terrae* is shown by arrows. Images were taken at bar 200 μm (**a**), bar 20 μm (**b**), and bar 10 μm (**c** and **d**)

*Nevskia terrae* had colonized the cotton paper, according to SEM images (Fig. 11a–d). The walls of the cotton fibers were plainly seen to be eroding and degrading, as depicted in Fig. 11a, b. High magnification revealed the bacterial spores covering the cotton fiber walls (Fig. 11c, d).

#### Nevskia terrae growth suppression on cotton paper treated with the biofilms

**Fig. 12:**
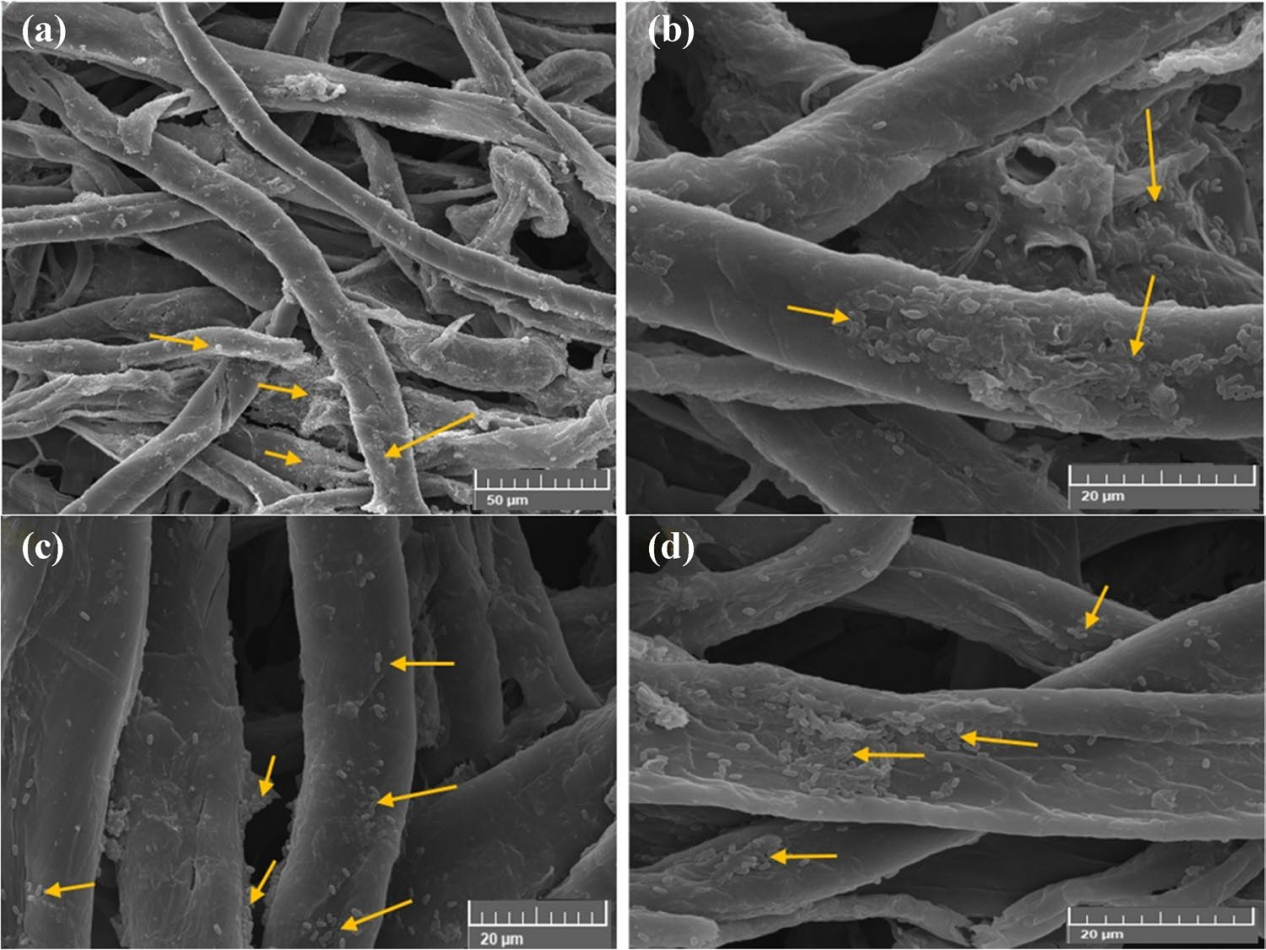
Inhibition patterns of *N. terrae* on cotton paper when treated with CMC/PGP poultice. The growth development of *N. terrae* is shown by arrows. Images were taken at bar 50 μm (**a**), bar 20 μm (**b**, **c**, and **d**)

Figure 12a–d displays the CMC/PGP formulation-treated cotton fibers that had no effect on *N. terrae* growth. Under the SEM, the CMC/PGP formulation had an impact on the cotton fibers, causing some fissures to be seen and deposits of poultice to be discovered on the fibers, which revealed a significant proliferation of *N. terrae* bacilli.

**Fig. 13:**
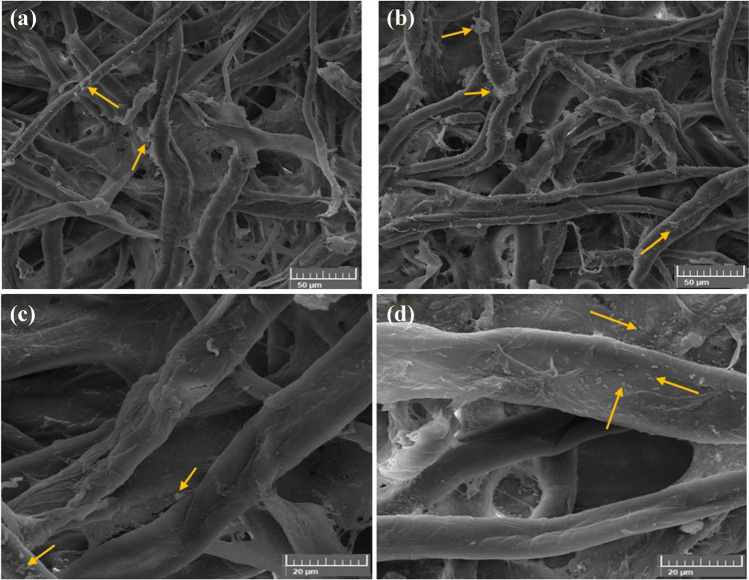
Inhibition patterns of *N. terrae* on cotton paper when treated with CMC/PGP/TiO_2_-1 poultice. The growth development of *N. terrae* is shown by arrows. Images were taken at bar 50 μm (**a**, and **b**), and bar 20 μm (**c**, and **d**)

Figure 13a–d shows the cotton paper that had been infected with *N. terrae* and treated with CMC/PGP/TiO_2_-1. Cotton fiber treated with CMC/PGP/TiO_2_-1 resulted in fiber opening and the appearance of cracks and ruptures (Fig. 13a, b), but it also affected the bacterial cell, resulting in a change in the bacterial cell’s morphology(Fig. 13c, d).

**Fig. 14:**
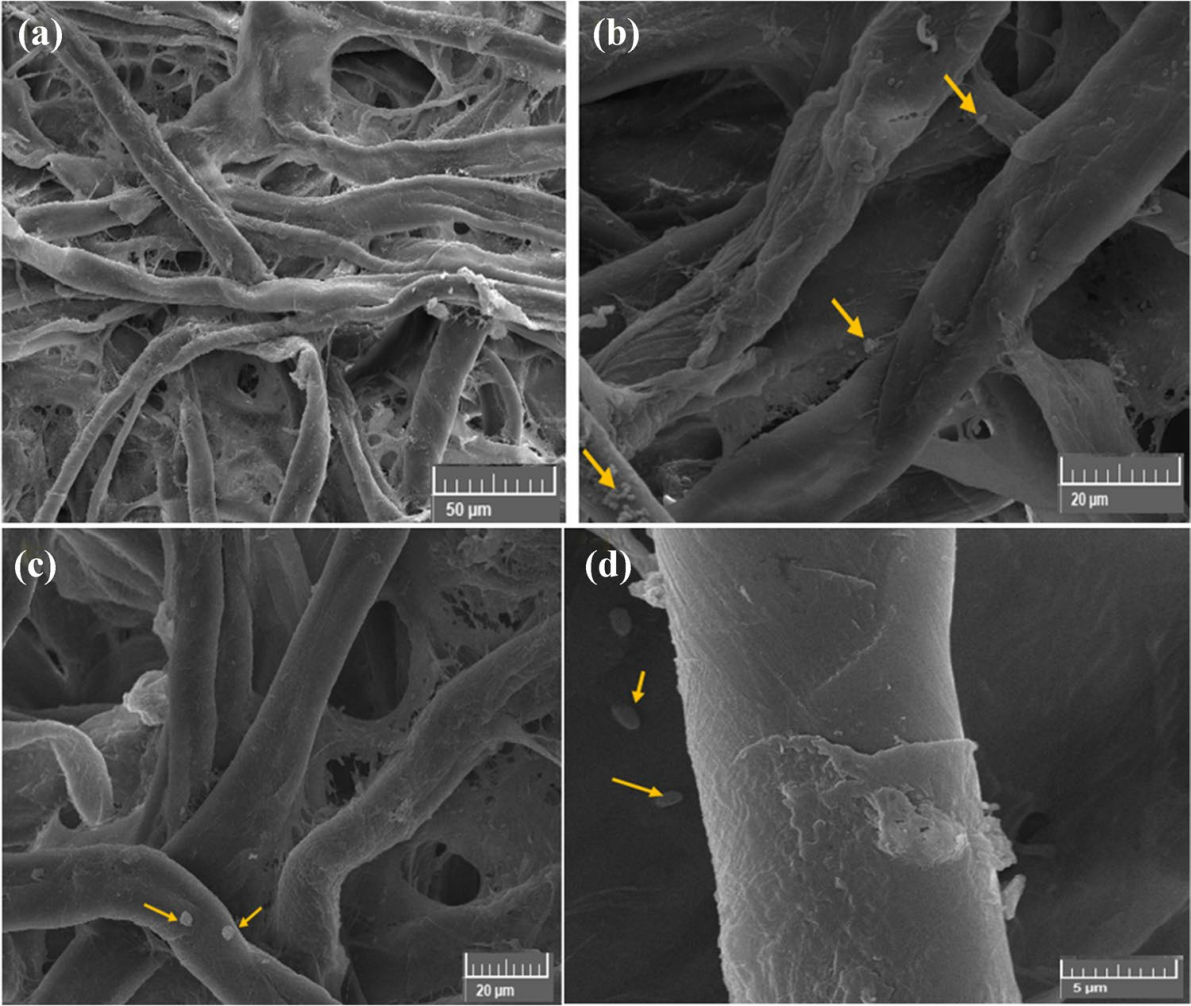
Inhibition patterns of *N. terrae* on cotton paper when treated with CMC/PGP/TiO_2_-2 poultice. The growth development of *N. terrae* is shown by arrows. Images were taken at bar 50 μm (**a**), bar 20 μm (**b**, and **c**), and bar 5 μm (**d**)

The infected cotton fibers with *N. terrae* and the CMC/PGP/TiO_2_-2-treated poultice are shown in Fig. 14a–d. The cells of *N. terrae* are destroyed, and the bacilli of *N. terrae* are adsorbed across the fibers (Fig. 14c, d). Furthermore, nearly deposits are not discovered of the poultice on the fibers.

**Fig. 15:**
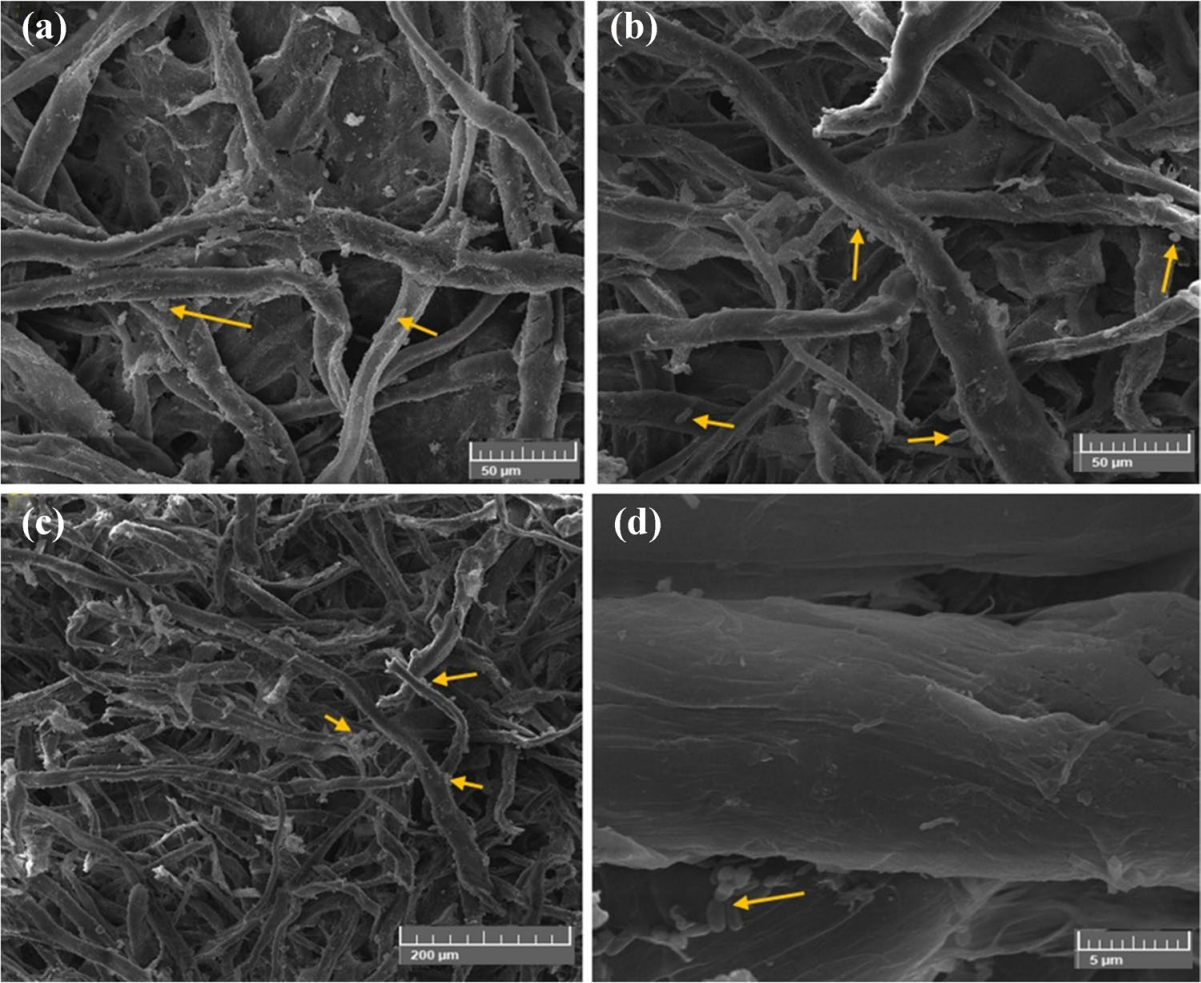
Inhibition patterns of *N. terrae* on cotton paper when treated with CMC/PGP/TiO_2_-3 poultice. The growth development of *N. terrae* is shown by arrows. Images were taken at bar 50 μm (**a**, and **b**), bar 200 μm (**c**), and bar 5 μm (**d**)

Figure 15a–d displays the *N. terrae*-infected cotton fibers that were poulticed with CMC/PGP/TiO_2_-3. It was evident that there are a few tiny gaps in the cotton fiber’s structure, which may be caused by the natural, non-homogeneous coating of fibers during the manufacturing process (Fig. 15c, d).

Based on the aforementioned findings, new natural nanocomposite polymer formulations were created and tested as poultices for cleaning paper from bacterial and fungal contamination. Because of the unique characteristics of TiO_2_NPs, including antibacterial and photocatalytic performance, it is one of the most extensively investigated materials in the field of antimicrobial activities. TiO_2_NPs typically exhibit all of the physical effects associated with absorption, reflection, and scattering of light when exposed to light with a bandgap energy of 3.2 eV or greater (Xie and Hung 2019). The extremely reactive hydroxyl radical (OH•), which is created when the holes react with the water in the air, is created as an electron donor. The superoxide ion is formed when oxygen, which continually exists on the surface of the particles, serves as an electron acceptor. Superoxide ions, hydroxyl radicals, and holes are all highly effective oxidants that can be utilized to oxidize and break down organic materials including odor molecules, viruses, and bacteria into water and carbon dioxide (Othman et al. 2014).

Reactive oxygen species (ROS) are essential for bioactivity, but the precise and reliable underlying mechanisms for signal processing, signal integration, and other signaling pathways, like NO, are still developing. Redox, oxidative, ion, and hormonal homeostasis are related to NO crosstalk functions through signaling pathway modification of downstream genes (Mariyam et al. 2023). Therefore, the generation of reactive oxygen species (ROS), which have numerous effects on bacterial cells that result in their death (Verdier et al. 2014), is attributed to the antibacterial action of TiO_2_NPs. Additionally, due to their tiny size, TiO_2_NPs can penetrate cell walls and membranes, enhancing intracellular oxidative damage. For instance, they can lessen DNA damage, H_2_O_2_ accumulation, and Cr(VI) accumulation (Kumar et al. 2023).

Numerous studies that used TiO_2_NPs in various formulations have shown potential antimicrobial benefits as well as improved the properties of the coated materials (Younis et al. 2023). Incorporating TiO_2_NPs into polyurethane matrices is a successful method for enhancing both the physicochemical and antimicrobial properties of polyurethanes (Saleemi and Lim 2022). Polypropylene films coated with TiO_2_NPs inhibited the growth of *E. coli* (Chawengkijwanich and Hayata 2008). TiO_2_NPs/chitosan nanocomposites incorporated in the CMC adhesive prevent the yellowness of CMC adhesive and enhanced the antifungal activity against *Aspergillus flavus* and *A. niger* (Ariafar et al. 2018). CMC/gelatin/TiO_2_–Ag nanocomposite formulation showed a very important antimicrobial activity against *E. coli* and *S. aureus* (Pirsa et al. 2020). Coated cotton fabrics with CMC/polyvinyl alcohol (PVA)/TiO_2_ nanocomposites and exposed to gamma irradiation showed potent antibacterial activity against *E. coli* (Khafaga et al. 2016).

These formulations included CMC and PGP, which were combined with TiO_2_NPs. CMC has been used because of its accessibility, low cost, simple pulping process, and superior film-forming capabilities with a very good bond between papers (Baker 1984; Mazhari Mousavi et al. 2017). It has been reported that the CMC coating could simultaneously improve the mechanical and water vapor barrier properties of paper materials (Basta et al. 2015).

Cellulose nanocrystals (CNC)-immobilized AgNPs (CNC@AgNPs) were synthesized and formulations of CMC/CNC@AgNPs used to coat paper surface enhanced mechanical and barrier properties and excellent antibacterial activities against *Escherichia coli* and *Staphylococcus aureus* (He et al. 2021). CMC/polyamideamine epichlorohydrin (PAE) interactions showed good wet and dry tensile strength of PAE-based handsheets of papers (Siqueira et al. 2015). Under both wet and dry conditions, tensile strength of cellulose fiber networks was significantly improved as reinforced with CMC/chitosan complex layer-by-layer (Wu and Farnood 2014). Anionic CMC-formed polyelectrolyte polymer (PEC) complex with cationic antimicrobial-wet strength polymer inhibited the growth of *E. coli* by destroying its cell membrane and causing the leakage of intracellular components from cells (Qian et al. 2008). The prepared AgNPs/CMC-layered double hydroxide (Ag/CMC-LDH) nanocomposite hydrogels were observed to have potential antibacterial activity against *E. coli* and *S. aureus* (Yadollahi et al. 2015). Coating a wrapping paper with the biopolymer, a ternary blend of carbohydrates (alginate, CMC, carrageenan) and grapefruit seed extract, significantly increased tensile properties of the paper and strong antibacterial activity against *Listeria monocytogenes* and *E. coli* (Shankar and Rhim 2018). AgNP/CMC sample showed promising antibacterial properties toward *E. coli* (Basuny et al. 2015).

Mixtures of CuNPs with a consolidant (tetraethyl orthosilicate, methylethoxy polysiloxane, Paraloid B72, tributyltin oxide, or dibutyltin dilaurate) and a water-repellent hold were greatly promised for preventing re-colonization of stone after conservation treatment (Pinna et al. 2012). Microalgae-based biopolymer as a potential bioactive film showed the highest fungal growth inhibition against *Fusarium verticillioides* (70.2%) and *Fusarium* sp. (61.4%) at 2.24 mg/mL (Morales-Jiménez et al. 2020). ZnONP/polylactic acid coating layer for packaging application indicates antimicrobial activity against *E. coli* and *S. aureus* (Zhang et al. 2017). CMC/AgNPs showed to be effective in inhibiting the growth of *S. aureus* and *E. coli* (Li et al. 2018).

Paper strength properties can be improved by strength additives such as synthetic and non-biodegradable polymers most of which pose great health and environmental hazards (Bhardwaj et al. 2019). Biopolymer additives in the present work showed to enhance the mechanical properties of the produced poultices. Cationic starch forms a natural affinity with the cellulosic fibers resulting in more fiber–fiber interactions (Fatehi et al. 2010). Chitosan is a biodegradable, non-toxic, antibacterial as well as renewable commodity with potential application as a strength additive in papermaking (Habibie et al. 2016). It consists of basic amino groups due to which it becomes cationic in nature that allows better reaction with cellulosic pulp (Rinaudo 2006; Habibie et al. 2016). Coating of paper sheets with nanocellulose/chitosan formulations enhanced their mechanical and air permeability properties and antibacterial power has been obtained against *Salmonella*, *Staphylococcus aureus*, and *Pseudomonas aeruginosa* (El-Samahy et al. 2017). TiO_2_ is a desirable material for preserving CMC films and its treated papers, but its problem is that its restricted reactivity in the UV wavelength limits its use in indoor circumstances (Ariafar et al. 2018).

Finally, the addition of various volumes of TiO2NPs increased the efficiency of the produced natural biopolymers, carboxymethyl cellulose, and Phytagel plant cells. Additionally, covering the fibers with the material loaded on the poultice resulted in the preservation of the fibers, demonstrating that CMC/PGP/TiO_2_-2 is one of the greatest poultices used in terms of blocking the microbe and protecting the fibers. In addition to being easier to apply, eco-friendly treatments are better at sterilizing microorganisms. The created biofilm membranes could be employed as an alternate, environmentally friendly method of manuscript preservation.

## Conclusion

This study prepared biofilms of carboxymethyl cellulose (CMC) and Phytagel pant cell (PGP) loaded with three volumes of titanium dioxide nanoparticles (TiO_2_NPs) and compared them to the control sample (CMC/PGP). The surface structure of the CMC/PGP films changed from smooth to rough as a result of the incorporation of TiO_2_NPs. The produced films’ morphological features were flawless. The use of TiO_2_NPs with 1 mL CMC/PGP/TiO_2_-1 also increased the biofilm’s tensile strength. The produced biofilms’ tensile strength decreased as the volume of TiO_2_NPs increased (CMC/PGP/TiO_2_-2 and CMC/PGP/TiO_2_-3). However, it was shown that adding TiO_2_NPs at a sufficient volume reduced the produced films’ swelling percentage. However, it was shown that adding TiO_2_NPs at a sufficient volume reduced the produced films’ swelling percentage. In comparison to CMC/PGP/TiO_2_-2 and CMC/PGP/TiO_2_-1, CMC/PGP/TiO_2_-3 showed more edema. In terms of preventing the growth of *A. sydowii* and *N. terrae* while clearing the treated cotton paper of the bacteria, the biofilm poultice CMC/PGP/TiO_2_-2 showed the best results. Composite films demonstrated impressive antibacterial properties against microbial development and could be employed as a different, environmentally friendly method of manuscript preservation.

### Supplementary Information

Below is the link to the electronic supplementary material.

Supplementary file1 (DOCX 1116 KB)

## Data Availability

Data are available in the text of the manuscript.
