# Supplementary material for: Poultices as biofilms of titanium dioxide nanoparticles/carboxymethyl cellulose/Phytagel for cleaning of infected cotton paper by Aspergillus sydowii and Nevskia terrae
Source: Environ Sci Pollut Res Int. 2023 Oct 21;30(53):114625–45. doi: 10.1007/s11356-023-30353-7 (PMC10663203; doi:10.1007/s11356-023-30353-7)

**Poultices as biofilms of titanium dioxide nanoparticles/carboxymethyl cellulose/phytagel for cleaning of infected cotton paper by *Aspergillus sydowii* and *Nevskia terrae***

**Maisa M. A. Mansour^a^, Mohamed Z. M. Salem^b,^***

*^a^ Conservation Department, Faculty of Archaeology, Cairo University, Giza, 12613, Egypt*

*^b^ Forestry and Wood Technology Department, Faculty of Agriculture (El-Shatby), Alexandria University, Alexandria, 21545, Egypt*

*** Corresponding author:** [mohamed-salem@alexu.edu.eg](mailto:mohamed-salem@alexu.edu.eg)

**Figure S1** Fragment sequence for sequencing the isolated fungus

TGCGAGGCATGCCTCCGGGCGCCCAACCTCCCACCCGTGAATACCTAACACTGTTGCTTCGGCGGGGAACCCCCTCGGGGGCGAGCCGCCGGGGACTACTGAACTTCATGCCTGAGAGTGATGCAGTCTGAGTCTGAATATAAAATCAGTCAAAACTTTCAACAATGGATCTCTTGGTTCCGGCATCGATGAAGAACGCAGCGAACTGCGATAAGTAATGTGAATTGCAGAATTCAGTGAATCATCGAGTCTTTGAACGCACATTGCGCCCCCTGGCATTCCGGGGGGCATGCCTGTCCGAGCGTCATTGCTGCCCATCAAGCCCGGCTTGTGTGTTGGGTCGTCGTCCCCCCCGGGGGACGGGCCCGAAAGGCAGCGGCGGCACCGTGTCCGGTCCTCTAGCGTATGGGGCTTTGTCACCCGCTCGAGGAGGGCCGGC

**
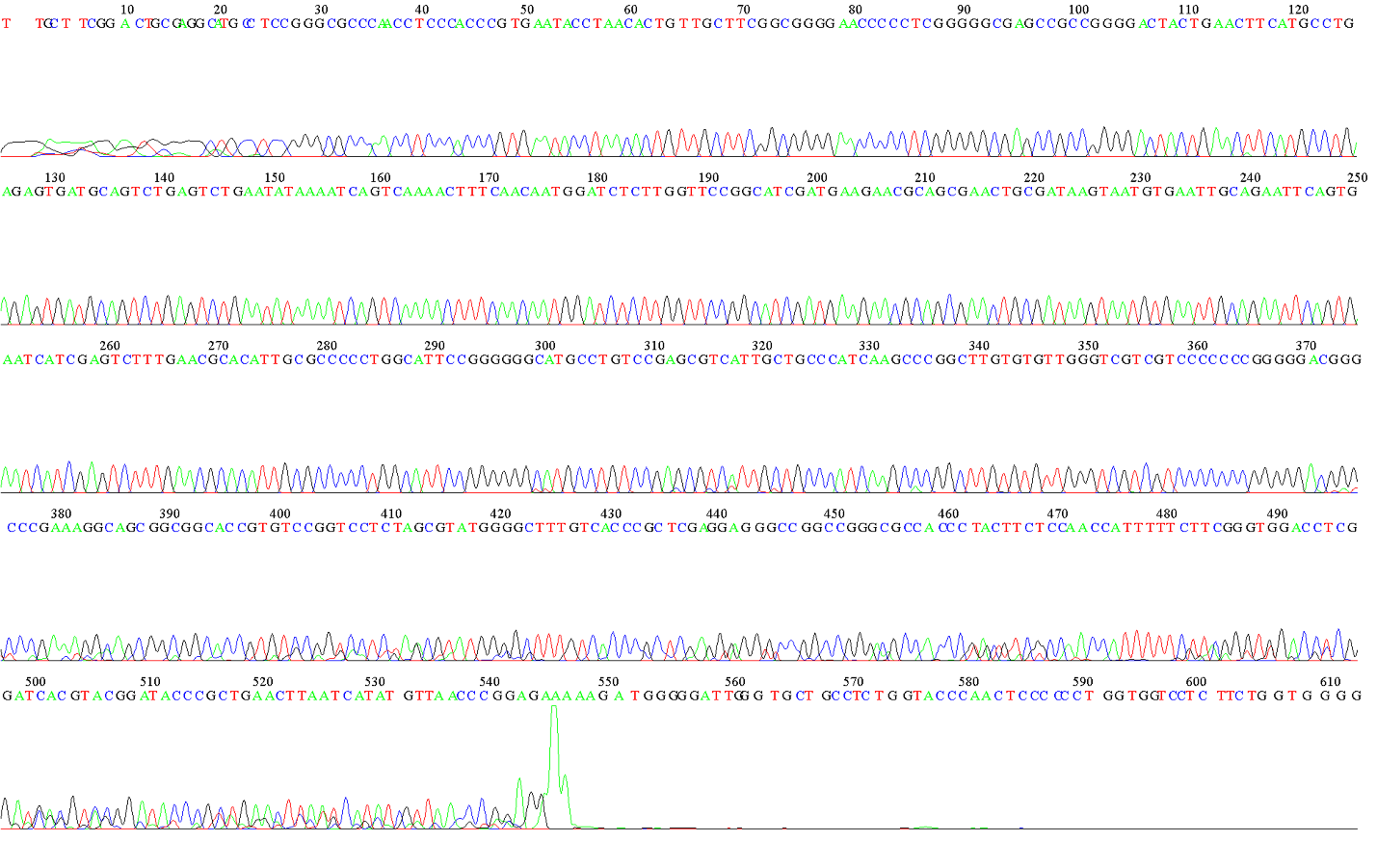
**

**
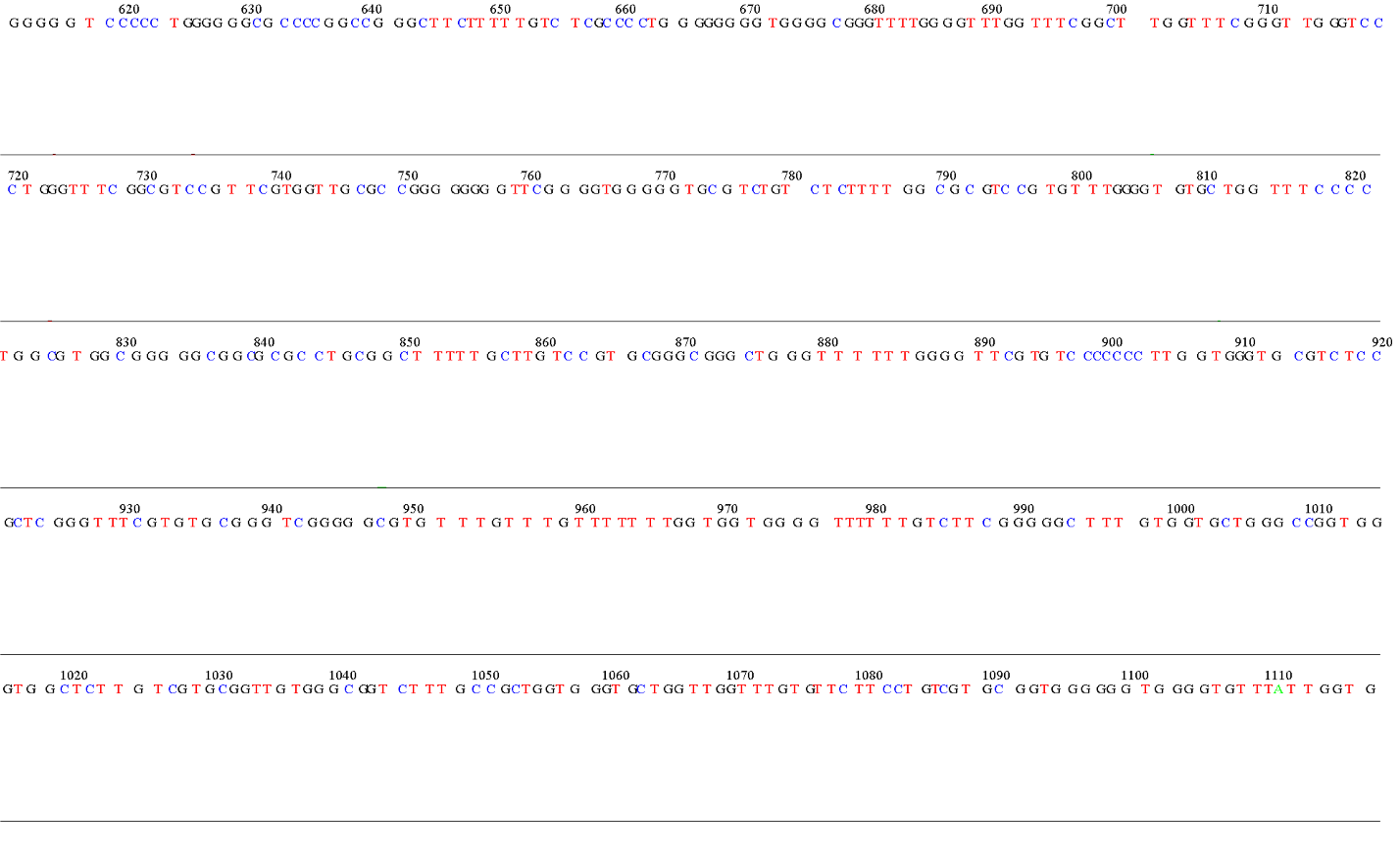
**

**Figure S2** Fragment sequence for sequencing for the isolated bacterium

AACGGGAGTACAATACTCTAGTGGCGGACGGGTGAGGAATACGTAGGAATTTGCCCTTAAGTGGGGGATAACCCGGGGAAACCCGGATTAATACCGCATGACCTCGAAAGAGCAAAGTGGGGGATCGCAAGACCTCACGCTTTTGGAGAAGCCTACGTCGGATTAGCTAGTTGGTGAGGTAATGGCTCACCAAGGCGACGATCCGTAACTGGTCTGAGAGGATGATCAGTCACACCGGAACTGAGACACGGTCCGGACTCCTACGGGAGGCAGCAGTGGGGAATATTGGACAATGGGCGCAAGCCTGATCCAGCAATGCCGCGTGTGTGAAGAAGGCCTTCGGGTTGTAAAGCACTTTAAGTTGGGAAGAAAAAACTCGCTCTAACACAGTGAGTCTTGACGGTACCGACAGAATAAGCACCGGCTAACTCTGTGCCAGCAGCCGCGGTAATACAGAGGGTGCAAGCGTTAATCGGATTTACTGGGCGTAAAGCGTGCGTAGGCGGTTCGTTAAGTCGATTGTGAAAGCCCTGGGCTTAACCTGGGAATTGCAGTCGATACTGGCGGACTAGAGAACGGTAGAGGGAGGCGGAACTCCAGGTGTAGCGGTGAAATGCGTAGATATCTGGAAGAACACCGATGGCGAAGGCAACCTCCTGGGCCTGTTCTGACGCTGAGGCACGAAAGCGTGGGTAGCAAACAGGATTAGATACCCTGGTAGTCCACGCTGTAAACGATGAGAACTTGACGTCGGGTCGCTTAGCGATTCAGTGTCGAAGCTAACGCGCTAAGTTCTCCGCCTGGGGAGTACGGCCGCAAGGTTGAAACTCAAAGGAATTGACGGGGGCCCGCACAAGCGGTGGAGTATGTGGTTTAATTCGATGCAACGCGAAGAACCTTACCTGGTCTTGACATGTCGAGAAGTCTGCAGAGATGCGGATGTGCCTTCGGGAACTCGAACACAGGTGCTGCATGGCTGTCGTCAGCTCGTGTCGGGAGATGTTGGGTTAAGTCCCGCAACGAACGCAACCCTTGCCCTTAGTTGCCATCATTCAGTTGGGCACTCTAAGG


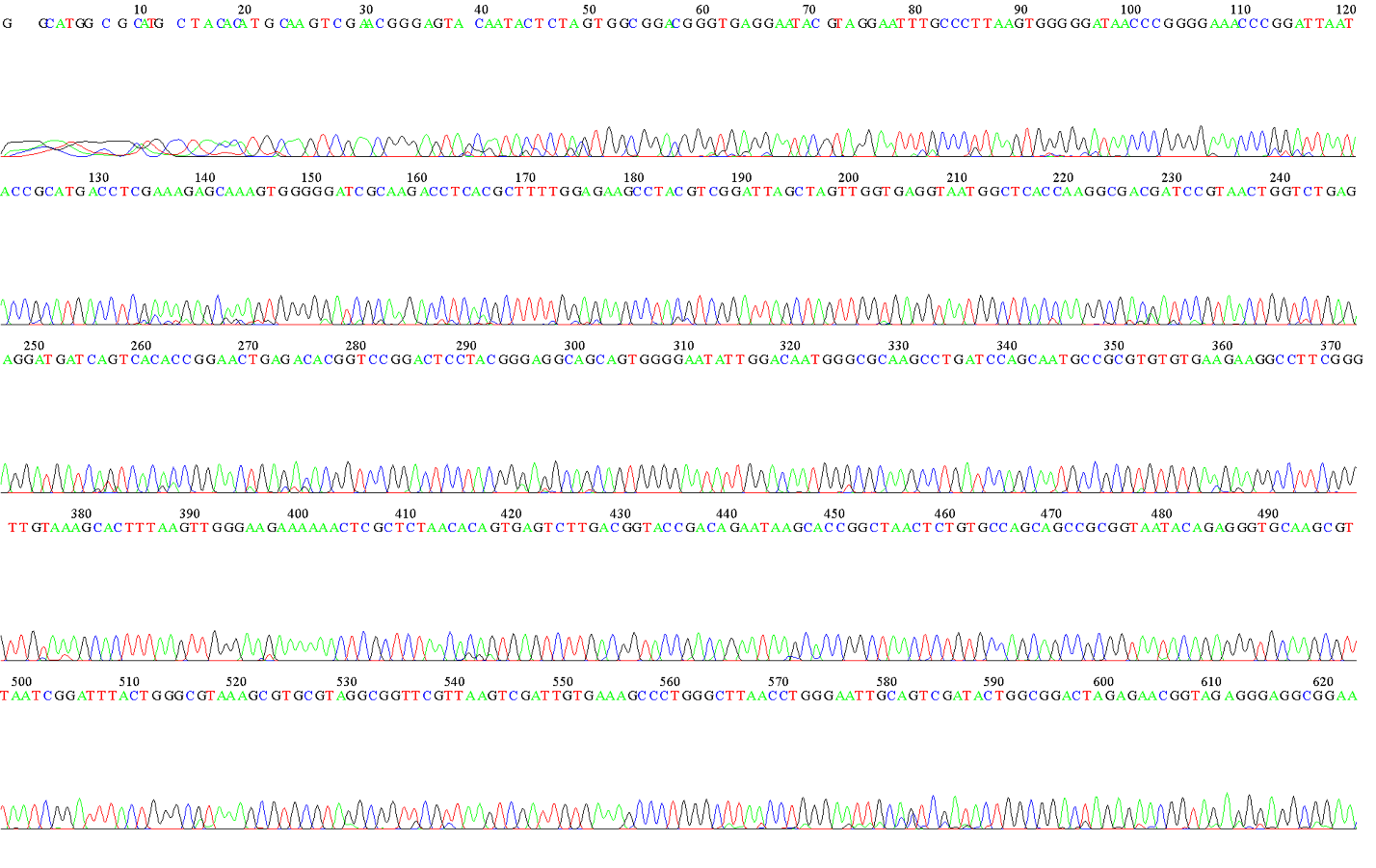


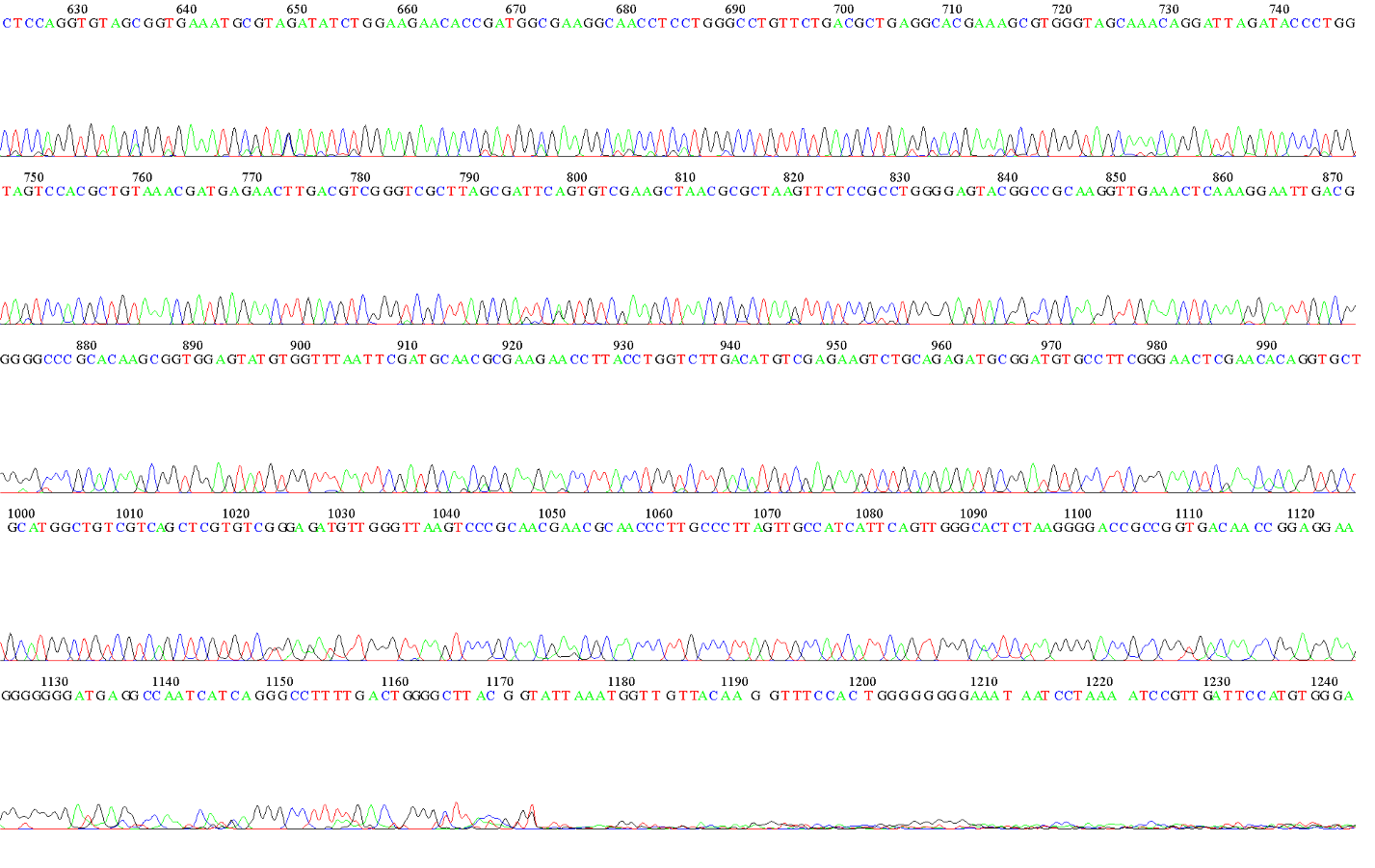


**Figure S3** Phylogenetic analysis tree of *Aspergillus sydowii* (MG991624)


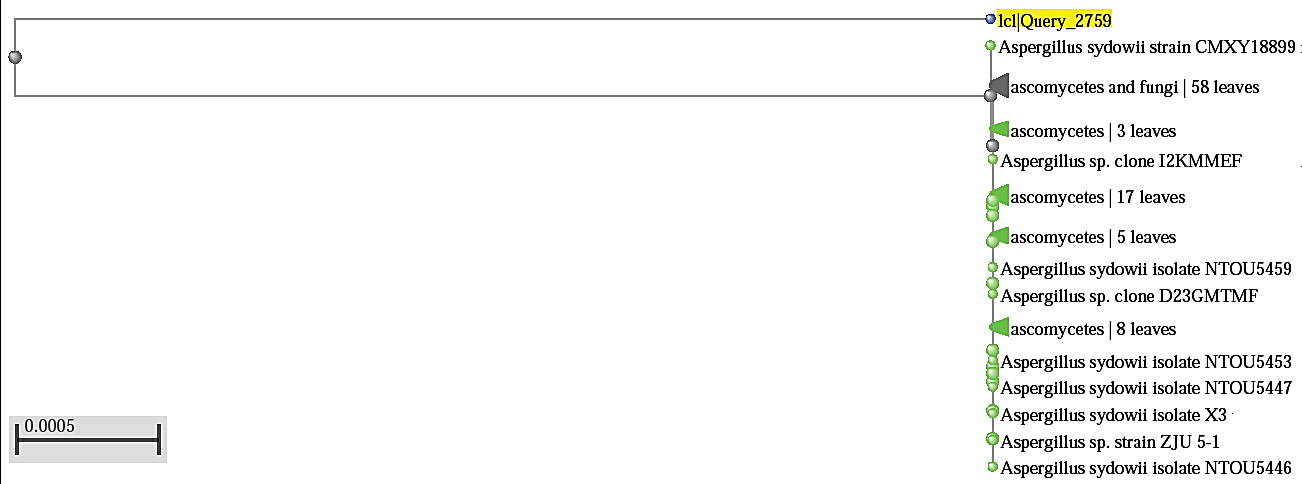


**Figure S4** Phylogenetic analysis tree of the uncultured bacterium *Nevskia terrae* (MG991624)


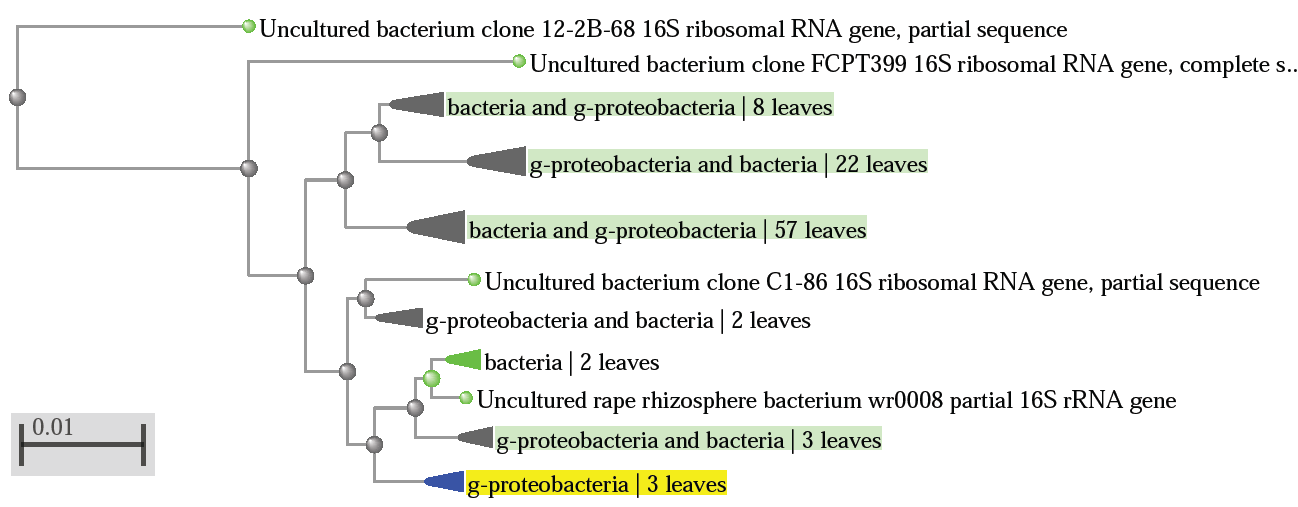

Supplement: Supplementary file 1 — Supplementary file1 (DOCX 1116 KB) [file 11356_2023_30353_MOESM1_ESM.docx]
